# Environmental and physiological factors shape the gut microbiota of Atlantic salmon parr (*Salmo salar* L.)

**DOI:** 10.1016/j.aquaculture.2016.07.017

**Published:** 2017-01-20

**Authors:** Carola E. Dehler, Christopher J. Secombes, Samuel A.M. Martin

**Affiliations:** Institute of Biological and Environmental Sciences, University of Aberdeen, Tillydrone Avenue, Aberdeen AB24 2TZ, UK

**Keywords:** Atlantic salmon, Gut microbiota, Next-generation sequencing

## Abstract

Gut microbes are key players in host immune system priming, protection and development, as well as providing nutrients to the host that would be otherwise unavailable. Due to this importance, studies investigating the link between host and microbe are being initiated in farmed fish. The establishment, maintenance and subsequent changes of the intestinal microbiota are central to define fish physiology and nutrition in the future. In fish, unlike mammals, acquiring intestinal microbes is believed to occur around the time of first feeding mainly from the water surrounding them and their microbial composition over time is shaped therefore by their habitat.

Here we compare the distal intestine microbiota of Atlantic salmon parr reared in a recirculating laboratory aquarium with that of age matched parr maintained in cage culture in an open freshwater loch environment of a commercial fish farm to establish the microbial profiles in the gut at the freshwater stage and investigate if there is a stable subset of bacteria present regardless of habitat type. We used deep sequencing across two variable regions of the 16S rRNA gene, with a mean read depth of 180,144 ± 12,096 raw sequences per sample. All individual fish used in this study had a minimum of 30,000 quality controlled reads, corresponding to an average of 342 ± 19 Operational Taxonomic Units (OTUs) per sample, which predominantly mapped to the phyla *Firmicutes*, *Proteobacteria*, and *Tenericutes*.

The results indicate that species richness is comparable between both treatment groups, however, significant differences were found in the compositions of the gut microbiota between the rearing groups. Furthermore, a core microbiota of 19 OTUs was identified, shared by all samples regardless of treatment group, mainly consisting of members of the phyla *Proteobacteria*, *Bacteroidetes* and *Firmicutes*. Core microbiotas of the individual rearing groups were determined (aquarium fish: 19 + 4 (total 23) OTUs, loch fish: 19 + 13 (total 32) OTUs), indicating that microbe acquisition or loss is occurring differently in the two habitats, but also that selective forces are acting within the host, offering niches to specific bacterial taxa.

The new information gathered in this study by the Illumina MiSeq approach will be useful to understand and define the gut microbiota of healthy Atlantic salmon in freshwater and expand on previous studies using DGGE, TGGE and T-RFPL. Monitoring deviations from these profiles, especially the core microbes which are present regardless of habitat type, might be used in the future as early indicator for intestinal health issues caused by sub optimal feed or infectious diseases in the farm setting.

**Statement of relevance:**

The Microbiome is central to gut health, local immune function and nutrient up take. We have used deep sequencing approach to show differences in rearing conditions of Atlantic salmon. This work is of interest to aquaculture nutritionists.

## Highlights

• Atlantic salmon (*Salmo salar*) parr harbour a vast diversity of microbiota in their distal intestine digesta.
• A core microbiota shared by all samples and accessory group core microbiotas were identified.
• Both rearing groups showed distinct intestinal microbiota profiles, with unique group-specific microbes.
• Maintenance of intestinal microbiota is a complex process, dictated by both environmental factors and host physiology.

## 1 Introduction

The gut microbiota can be viewed as an “extra organ” due to its key role in intestinal development, homeostasis and protection and it is now becoming clear that the microbiota has major influences on growth, health and development in all vertebrates (O'Hara and Shanahan, 2006, Dhanasiri et al., 2011). The host may also exert direct control on community assembly by physiological and lifestyle forces, offering a niche for specialised bacteria that are beneficial for their dietary life style (Roeselers et al., 2011). In return, intestinal microbes supply the host with exogenous nutrients and extracellular enzymes, fatty acids and vitamins which otherwise would not be available to the host (Dhanasiri et al., 2011). Hence a balanced microbial community is central to the wellbeing of animals and humans. An established microbiome also can reduce the proliferation of pathogenic bacterial species and as such are central to ensuring disease control (Austin, 2006, Fjellheim et al., 2007). As intestinal microbiota of fish play a key role in health, understanding the interactions between fish and gut microbiota is an important research field for fish health and aquaculture (van Kessel et al., 2011). Initial studies have shown that fish harbour a diverse microbiota, dependent on host species (Sullam et al., 2012, Li et al., 2014, Givens et al., 2015), life cycle stage (Giatsis et al., 2014, Ingerslev et al., 2014a, Zarkasi et al., 2014, Zarkasi et al., 2016) and diet (Navarrete et al., 2013, Wong et al., 2013, Ingerslev et al., 2014b). Fish present a unique case among vertebrates with microorganisms from environmental sources potentially having a much higher influence on bacterial composition and health status due to constant contact with water (Austin, 2006, Ringø et al., 2010).

The fish intestine can harbour 10^7^ to 10^11^ bacteria/g intestinal content, with aerobes and facultative anaerobes being more abundant than obligate anaerobes, and can be split between the autochthonous species that are attached to the intestinal mucosa and the allochthonous that do not attach either due to lack of ability or outcompetition by mucous attached bacteria (Nayak, 2010, Navarrete et al., 2012, Llewellyn et al., 2014, Givens et al., 2015).

Gut microbiota are detectable 1 day post hatching in rainbow trout, before first feeding commences, potentially due to contact with surrounding water and yok sac digestion (Ingerslev et al., 2014a). First feeding induces significant changes in the gut microbiota composition and higher transcription levels for immune system associated genes (Ingerslev et al., 2014a). Non-attached bacterial levels may be maintained artificially high in fish due to the constant contact with the surrounding water (Ringø et al., 2010). Genetic background and ontogenic factors are also factors that shape an individuals' gut microbiota (Benson et al., 2010, Ingerslev et al., 2014a). Previous studies showed that fish harbour more specialised gut microbiota than previously assumed (Abid et al., 2013). Furthermore, fish are able to retain specific bacteria, making gut microbial assemblages not a passive collection of seeding communities (Sullam et al., 2012).

Although intestinal microbiota profiles were found to cluster with their respective environmental water bacterial profiles, many bacterial species found in the intestine where not detected at all in the environmental water samples, indicating maintenance of key species derived from elsewhere (Roeselers et al., 2011, Sullam et al., 2012). This suggests that a large percentage of gut microbiota may actually be specialised members of symbiotic communities rather than free-living microbes (Sullam et al., 2012).

Recent technological advances in sequencing have led to a much more comprehensive assessment of the intestinal microbiota. Studies until a few years ago relied on either growing bacteria *ex vivo*, for which only very small numbers of species could be assessed or using techniques such as DGGE and TGGE. Only about 1% of all gut bacteria in fish can be cultured, including *Enterobacteriaceae*, *Pseudomonas* spp., *Staphylococcus* spp., *Acinetobacter* spp., *Aeromonas* spp., *Vibrio* spp. and *Bacillus* spp., which severely limited early assessments of fish intestinal microbiota (Merrifield et al., 2009), with a limited number of species cultivated from zebrafish (Cantas et al., 2012) and trout (Skrodenyté-Arbačiauskiené et al., 2008). In Atlantic salmon limited species richness was found by TTGE (Navarrete et al., 2009a) and DGGE (Reveco et al., 2014).

Next-generation sequencing platforms including Roches' 454 and Illumina MiSeq, NextSeq 500, and HiSeq 2000 have overcome the limitations of the previous technologies (Van Kessel et al., 2011, Geraylou et al., 2013, Star et al., 2013, Ingerslev et al., 2014a, Ingerslev et al., 2014b, Zarkasi et al., 2014). The 16S rRNA gene is characterised by highly conserved and highly variable regions across the whole sequence and has emerged as popular sequencing target, facilitating taxonomic assessments *via* the use of universal primers (Baker et al., 2003, Rajendhran and Gunasekaran, 2011, Klindworth et al., 2013). Read lengths of between 250 and 500 bp have been shown sufficient for community comparisons and current studies often sequence to a depth of > 100,000 sequences per biological sample (Liu et al., 2007, Hamady and Knight, 2009). Sequencing at such depth allows for rare species/genera to be identified from a number of key sequence resources designed for such studies, in particular RDP (Wang et al., 2007), SILVA (Pruesse et al., 2007) and Greengenes (DeSantis et al., 2006), all containing a massive resource of bacterial species 16S rRNA sequences.

In this study we investigated the role of environmental rearing conditions in the composition of intestinal microbiota in Atlantic salmon parr. Our aim was to define the microbiota of healthy Atlantic salmon at high resolution and investigate how important the rearing water conditions are to the maintenance of microbes in the fish intestine. To achieve this, fish were sampled from two different holding conditions: an indoor recirculating aquarium facility and a cage culture in an open loch environment. Both groups of fish came from the same hatchery before being transferred to the respective rearing conditions. We hypothesised that the fish in the open water environment would have a greater diversity of bacterial species in the intestine due to the natural variation of microbial composition in the water column caused by currents, rain water influx, over-land water run-off and greater biological species diversity, thereby offering a higher variety of potential colonisers. Alpha diversity analysis based on the Chao 1 index confirms the hypothesis, showing a significantly higher number of OTUs in the intestines of fish kept in the open loch system. We found 328 Operational Taxonomic Units (OTUs) only present in fish of the open loch system and 306 OTUs that were unique to the recirculating aquarium. Furthermore we identified 71 OTUs that were significantly different in abundance between the rearing groups, with 61 OTUs being more abundant in open loch system. Additionally we found a core microbiota within the rearing groups and overall, regardless of source of the fish. To our knowledge, this is the first in depth study of bacterial OTUs and core microbiota harboured in the intestine of Atlantic salmon parr at high sequencing depth.

## 2 Material and methods

### 2.1 Fish maintenance and sampling

Two groups of juvenile mixed sex Atlantic salmon were collected, one from open freshwater commercial cages on the West coast of Scotland and a second group from a recirculating aquarium system at the University of Aberdeen (n = 20 for each group). Both groups were derived from the same hatchery before being moved to the different rearing environments and were at the pre-smolt stage at the time of sampling. In the aquarium the temperature was kept at 12 °C, pH 7.6 under natural photoperiod and oxygen saturation of 90%. Fish were fed *ad libitum* by hand twice daily at 09:00 and 15:00 for eight months and feed rate was adjusted each month to ensure around 2% of their body weight was consumed per day. The fish in the open cage system were under natural ambient temperature and natural photoperiod. Both groups were fed a fish meal rich commercial diet (BioMar). Fish were killed by anaesthesia overdose (phenoxyethanol) followed by destruction of the brain. The final weights of the fish were 59.3 ± 9.2 g for the open loch group and 124.7 ± 33 g for the aquarium group. The digesta samples (100–200 mg) were collected in 1.5 ml Eppendorf tubes immediately following death and frozen on dry ice before longer term storage at − 80 °C.

### 2.2 DNA extraction

For DNA extraction, the QIAamp Fast DNA Stool Mini Kit (Qiagen) was used according to the manufacturers protocol with the following modifications. InhibitEx buffer was directly applied to the frozen digesta samples and two tungsten beads were added. The samples were then pre-treated with mechanical lysis by TissueLyser for 5 min to avoid biases against tough-walled Gram-positive bacteria. Lysis temperature was 90 °C to allow for cell-wall break-down of difficult to lyse bacteria. DNA was quantified by NanoDrop spectrometry.

### 2.3 PCR amplification and sequencing

For primary PCR reactions, variable regions 3 and 4 of the 16S rRNA gene were targeted with the recently published primer pair by Klindworth et al. (2013) including a 5′ overhang as suggested by Illumina. The forward primer had the sequence 5′ TCGTCGGCAGCGTCAGATGTGTATAAGAGACAG**CCTACGGGNGGCWGCAG**, and the reverse primer 5′ GTCTCGTGGGCTCGGAGATGTGTATAAGAGACAG**GACTACHVGGGTATCTAATCC** with the bold underlined sequence being the locus-specific V3-V4 primers. PCR reactions were performed in triplicate for each sample and pooled after amplification to avoid biases in PCR efficiencies. PCR reactions were performed in 25 μl reactions including 5 μl of each forward and reverse primer (1 μM each, Sigma), 12.5 μl of 2 × Kapa HiFi HotStart ReadyMix including high-fidelity polymerase (Kapa Biosystems) and 2.5 μl of DNA (5 ng/μl). PCR conditions included an initial denaturation at 95 °C for 3 min, followed by 25 cycles of 30 s at 95 °C, 30 s at 55 °C and 30 s at 72 °C after which a final extension at 72 °C for 5 min was applied. The product (527 bp, 460 bp + 67 bases of overhang primer) was verified for each PCR reaction on a 1.5% agarose gel.

These PCR products were cleaned with AMPure XP (Agencourt) and verification of clean-up product with TapeStation (Agilent) was performed. This was followed by indexing PCR which attached Nextera XT barcodes and Illumina sequencing adapters to the 5′ overhangs. After another round of AMPure XP (Agencourt) clean-up, multiplexed amplicons were sequenced with Illumina MiSeq from both ends.

### 2.4 Bioinformatic analysis

Raw forward and reverse sequences were assembled for each sample into contigs using mothur (Schloss et al., 2009). The assembled reads were then quality-filtered one sample at a time using the mother script trim.seqs, excluding reads that were < 400 bp long, had a lower Phred quality score average than 25, had a homopolymer run longer than 10 bases and nucleotide differences of > 7 bases in the primer region. The resulting individual fasta files were assembled into one fasta file and all further analysis was performed in QIIME (Caporaso et al., 2010). Operational Taxonomic Units (OTUs) were picked *de novo* using Uclust at the 97% similarity level. A reference set was picked randomly from the resulting OTU bins. Taxonomy was assigned using the RDP classifier (Wang et al., 2007) at an 80% confidence level. The resulting fasta file was summarised in an OTU table in biom format. Taxonomy-assigned sequences were aligned with PyNAST with a pairwise Uclust alignment algorithm against the Greengenes data base (Release GG_13_5, DeSantis et al., 2006). ChimeraSlayer was used to identify chimeric sequences, which were afterwards excluded from both OTU table and alignment. The known contaminant sequences of *Cyanobacteria* and singleton sequences were removed from the OTU table and cropped from the alignment. The final quality-filtered alignment was used to make a phylogenetic tree of the reference OTUs as a basis for alpha-diversity (diversity within treatment group) metrices. Shared OTUs between all samples and a core microbiome was computed and visualised in the Phyloseq package in R. Metastats (White et al., 2009) was used to identify which OTUs were significantly different between the environmental groups.

Alpha diversity metrics were calculated for Chao1 (assesses differences in species richness) and PD-whole-tree (assesses differences based on phylogenetic information). Rarefaction curves were computed for both metrics at sample and treatment level. The highest common rarefaction level of all samples was identified and used as the basis to test for statistical differences between aquarium and loch using Bonferroni-corrected non-parametric testing with 999 Monte-Carlo permutations. To assess beta-diversity (phylogenetic differences between treatment groups), distance matrixes were produced with an even sequence level at the highest common rarefaction level common for each sample. The distance matrixes were used to test for statistical differences between distances within and between treatments, both for weighted and unweighted UniFrac metrics and to produce Multiple dimension scale (MDS) plots (Lozupone and Knight, 2005, Lozupone et al., 2007). Visualisation of rarefaction plots and cluster analysis was done with the Phyloseq package in R (McMurdie and Holmes, 2013).

## 3 Results

### 3.1 Sequencing overview and microbiota characterisation

DNA was extracted from 30 fish, 15 from the aquarium and 15 from the loch. However, several samples showed poor PCR amplification, most likely due to co-extraction of PCR inhibitors, and were not used for sequencing, leaving 8 fish from the aquarium and 12 fish from the loch for analysis. In total, 3,602,871 raw reads were obtained for both forward and reverse directions after sequencing. The mean read depth per sample was 180,144 ± 12,069 sequences per read direction per sample. No reads were lost after assembly of forward and reverse reads. After the initial quality filtering, 3,226,813 sequences passed with a mean of 161,341 ± 12,472 sequences per sample. Across all samples a total of 51,591 OTUs were detected as defined by initial identification by the RDP classifier against the unaligned Greengenes database. Within these OTUs all singletons and *Cyanobacteria*, a commonly observed single cell algae, were removed, leaving 2153 OTUs. A further 303 OTUs were eliminated as these were identified as chimeric sequences, leaving a total of 1850 OTUs for subsequent analysis.

**Fig. 1:**
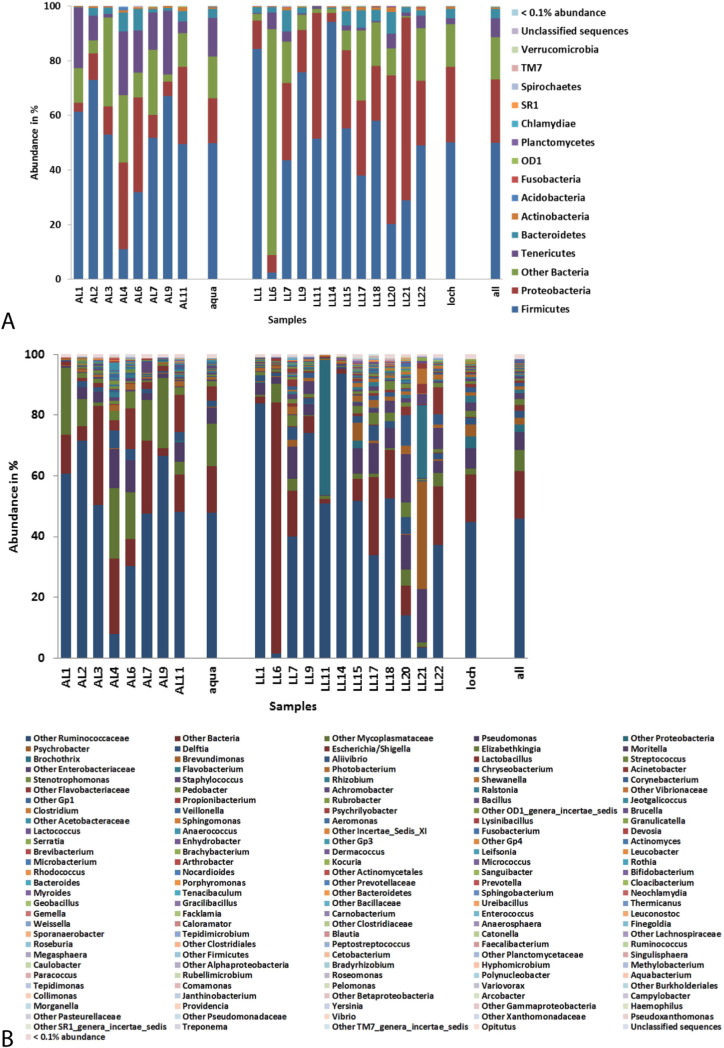
Relative abundance (%) of main bacterial taxa found in digesta collected from the distal intestine of Atlantic salmon (*Salmo salar*) parr at phylum level (A) and genus level (B). Phyla and genera below an abundance of 0.1% are not shown but summarised in a mixed group “< 0.1% abundance”. Bacterial profiles are shown on an individual level, a rearing group level (aqua = recirculating aquarium system, loch = open loch environment) and on an overall level (= all).

Overall, nine phyla were identified at an abundance ≥ 0.1% (Fig. 1A). The only difference in terms of presence/absence of phyla at a ≥ 0.1% abundance was that loch samples showed only 8 phyla, lacking *Acidobacteria* at this abundance level. When samples are investigated individually, 16 phyla were identified at an abundance ≥ 0.1%. The intestinal microbiota of the fish examined was dominated by *Firmicutes* (50%), *Proteobacteria* (23.1%) and *Tenericutes* (6.9%), accounting for 80% of all sequences. OTUs mapped to “other Bacteria” (representing 15.5%) reflect a mix of different phylogenetic groups that could not be identified to a deeper taxonomic level and are not considered a homogenous group.

When the OTUs are considered at the genus level, a high diversity of microbes is identified. Overall, 43 genera were identified at ≥ 0.1% abundance (Fig. 1B). Samples from the aquarium group harboured 39 genera and loch samples harboured 44 genera at ≥ 0.1% abundance. When samples are considered individually, the number of genera increases to 145 genera at ≥ 0.1% abundance, reflecting the high inter-individual variation in microbiota composition. Across all samples, the most abundant genera were classified as “Other *Ruminococcaceae*” (46%), followed by “Other *Mycoplasmataceae*” and *Pseudomonas* sp. (6.9% and 6.1%).

### 3.2 Microbiota differences between recirculated aquarium and open loch

**Fig. 2:**
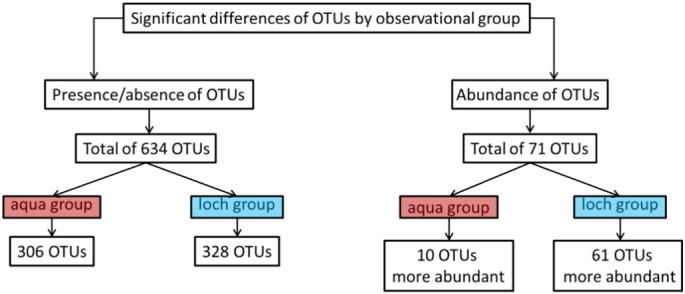
Operational Taxonomic Units (OTUs) of microbes identified in the distal intestine digesta of Atlantic salmon (*Salmo salar*) parr found to be significantly different between rearing groups (recirculating aquarium group and open loch environment group) by Metastats analysis (see Supplementary Tables 1 and 2). 634 OTUs were found to be entirely significantly present, of which 306 were significantly present in the aquarium group and 328 significantly present in the loch group. Furthermore 71 OTUs were found to show significantly different abundance between the observational groups, with 10 OTUs that were more abundant in the aquarium group and 61 OTUs that were more abundant in the loch group (see Supplementary Table 3). For a full list of OTUs found to differ significantly between observational groups refer to Supplementary Tables 1, 2 and 3.

To compare differences in gut microbial community composition between fish reared in the recirculating aquarium and the open loch, Metastats analysis was performed (Fig. 2). OTUs were identified that were present in either group at a statistically significant level with OTUs only present in the aquarium reared fish (Supplementary Table S1), OTUs only present in the loch reared fish (Supplementary Table S2) and OTUs significantly more abundant in either group (Supplementary Table S3).

Metastats analysis found that 306 OTUs were unique to the fish from the recirculating aquarium system, mapping to 16 phyla, and loch samples harboured 328 unique OTUs, mapping to 13 phyla. In samples from the aquarium, 68 OTUs could only be identified to “unclassified bacteria” (UB) (11 OTUs could not be assigned a Greengenes ID) and three OTUs to “unclassified sequence” (US), whereas in samples from the open loch, 99 OTUs could only be identified to UB (31 OTUs could not be assigned a Greengenes ID) and six OTUs to US. OTUs included in the phyla *Chloroflexi*, *Chloroplast*, *Tenericutes* and *Verrucomicrobia* were only found in samples from the recirculating aquarium, whereas OTUs included in the phylum *OP10* were only found in samples from the open loch.

A further 71 OTUs were significantly different in abundance between the environmental groups with higher abundance of 10 OTUs in fish from the aquarium and 61 OTUs more abundant in samples from the loch. Forty one OTUs could only be identified to an UB level, of which only one OTU had no match in the Greengenes database. Most OTUs with significant differences in abundance were associated with either the phylum *Firmicutes* (11 OTUs) or *Proteobacteria* (12 OTUs) (Supplementary Table S3).

### 3.3 Core microbiota

**Fig. 3:**
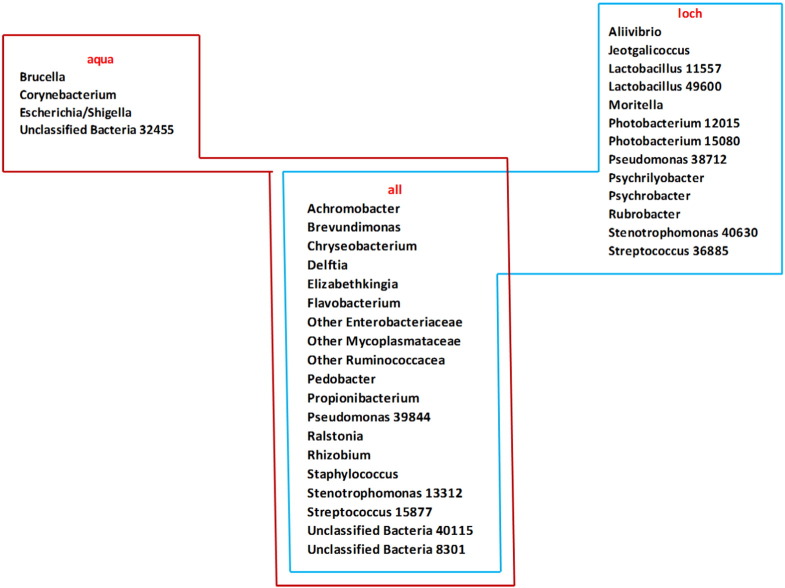
Core microbiota of distal intestine digesta in Atlantic salmon (*Salmo salar*) parr. Core microbiota was identified at an overall level (regardless of rearing group), and at a rearing group level (aqua = recirculating aquarium facility, loch = open loch environment).

The core microbiome across all individuals examined was 19 shared OTUs, including one *Actinobacteria*, for *Bacteroidetes*, three *Firmicutes*, eight *Proteobacteria*, one *Tenericutes* and two “Other” bacteria (Fig. 3). Within this core microbiota, dominant members like *Ruminococcaceae* spp., *Mycoplasmataceae* spp. and *Pseudomonas* spp. were found, but also genera of much smaller relative abundance, for example, *Chryseobacterium* spp. and *Staphylococcus* spp. were identified. When separated by treatment group, the aquarium fish shared a core microbiota of 23 OTUs, with four additional OTUs to the above overall core microbiota (*Escherichia/Shigella*, *Brucella*, *Corynebacterium* and one “Other Bacteria”), whereas loch fish harboured additional 13 OTUs (two *Photobacterium*, *Aliivibrio*, *Moritella*, *Psychrobacter*, *Jeotgalicoccus*, two *Lactobacillus*, *Psychrilyobacter* and *Rubrobacter*, plus an additional OTU for *Pseudomonas*, *Stenotrophomonas* and *Streptococcus*) representing a core microbiota of 32 OTUs.

### 3.4 Alpha diversity

Samples had an average of 342 ± 19 OTUs, with the lowest being AL4 harbouring 176 OTUs and the highest LL7 with 525 OTUs. The Chao1 index found that the number of OTUs was significantly different (*P* = 0.037) with a higher number of OTUs in the loch samples. The PD_whole_tree metric, however, showed no significant differences in phylogenetic diversity between the two rearing groups, indicating that the differences in microbial species richness are due to enrichments of the same phylogenetic branches in the loch rearing group, rather than addition of novel phylogenetic groups. To test for robustness on even sequence levels per sample, rarefaction curves were produced based on the Chao1 metric with an iteration of 10 per rarefaction level on 100 levels from 10 sequences/sample to 134,410 sequences/sample. The rarefaction curves reach a plateau between 20,000 and 30,000 reads and confirm the higher diversity of OTUs found in the loch group, showing that evening the sequencing depth to this level for all samples is enough to identify patterns unique to the rearing groups (data not shown).

Rarefaction analysis identified the highest common rarefaction level of all samples is 33,610 sequences/sample. This limitation arose from sample AL6. Hence, all samples were limited to 33,610 sequences/sample randomly picked to facilitate evenness for beta diversity comparisons.

### 3.5 Beta diversity

**Fig. 4:**
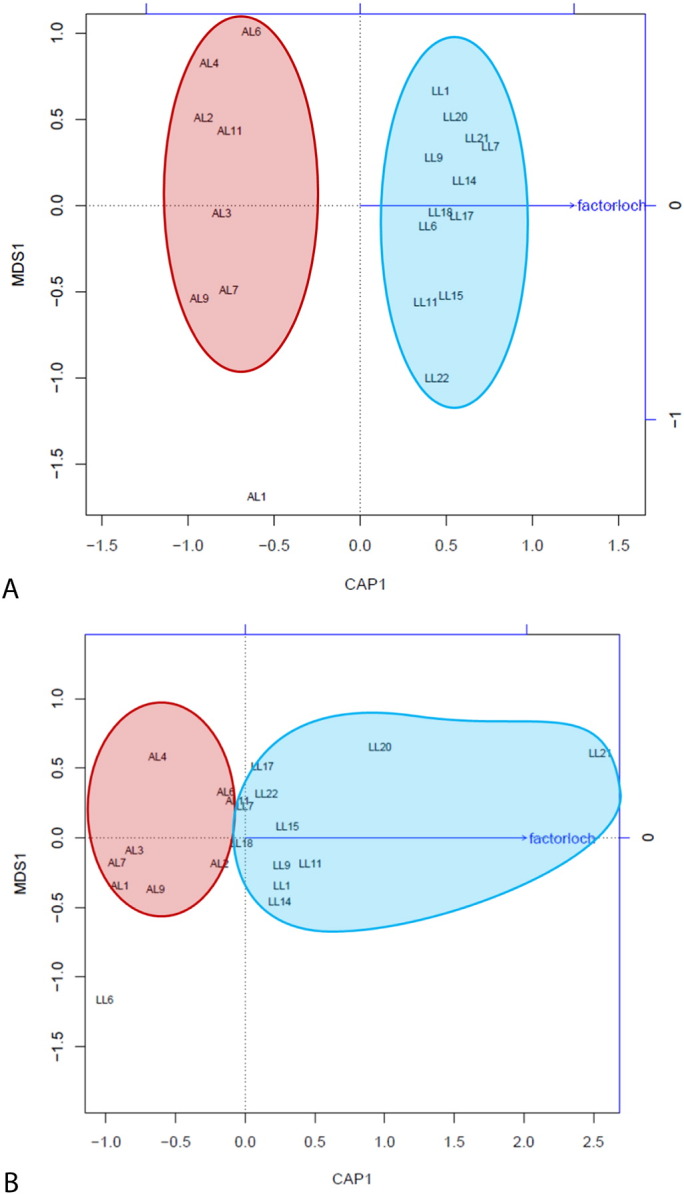
Multiple dimension scale (MDS) plots of unweighted (A) and weighted (B) UniFrac distances of distal intestine digesta microbiota found in Atlantic salmon (*Salmo salar*) parr by rearing group. Beta diversity analysis of unweighted UniFrac (A, presence/absence of Operational Taxonomic Units (OTUs)) was found significantly different between observational groups (PERMANOVA, Pseudo F-statistic 1.4599, *P* = 0.006, based on 999 permutations). However, analysis of weighted UniFrac (B, presence/absence/abundance of OTUs) was not found significantly different (PERMANOVA, Pseudo F-statistic 0.9589, *P* = 0.419, based on 999 permutations). Red = recirculating aquarium facility, blue = open loch environment.

Sample clustering was visualised with multiple dimension scaling (MDS) based both on unweighted and weighted UniFrac (Fig. 4A and B). Distance-based Redundancy Analysis (dbRDA) associated with MDS plots showed that based on the unweighted UniFrac distance matrix, there is a significant separation of the clusters by rearing group (*P* = 0.006). However, dbRDA performed on the weighted UniFrac distance matrix showed that there is no significant difference between the two rearing groups (*P* = 0.44). Additional statistical testing with PERMANOVA confirmed significant differences between the environments based on absence/presence of OTUs (Pseudo F-statistic 1.4599, *P* = 0.006), but not in terms of abundances of the OTUs (Pseudo F-statistic 0.9589, *P* = 0.419). The significance of the unweighted absence/presence approach reflects the results from the Metastats analysis, showing a high proportion of OTUs present in either one group or the other. The lack of significance seen in the weighted approach, which takes not only the absence/presence but also the abundance of each OTU into account, maybe due to the high individual variability of microbial composition masking the different patterns and introducing noise.

## 4 Discussion

The intestinal microbiota of animals and humans has attracted much recent attention as it is believed to be a key factor in numerous animal functions including health, growth and disease status (Ley et al., 2008, Hamady and Knight, 2009, Lee and Mazmanian, 2010, Kau et al., 2011, Gevers et al., 2012, Lozupone et al., 2012). Recent advances in deep sequencing technologies have made the detailed study of fish microbiota a relatively straight forward procedure and no longer relies on the ability to grow bacteria *ex vivo*, which meant studies were highly limited (Austin, 2006, Navarrete et al., 2009b Geraylou et al., 2013). In this study the intestinal microbiota of Atlantic salmon has been examined under two different environments, a recirculated aquarium facility and an open freshwater loch cage system in the West of Scotland using the 16S rRNA marker gene. Both groups of fish had been reared in a common flow through hatchery condition before being maintained in either the aquarium or loch. Our hypothesis was that a core microbiota would be maintained in both groups, but that the diversity might be altered by the environmental conditions.

The concept of a core set of microbial species fulfilling the minimal symbiotic functionality, has been suggested and promoted previously (Roeselers et al., 2011, Star et al., 2013). However, in humans, limited evidence has been given for universally abundant species at a fine taxonomic level (Lozupone et al., 2012). Recent studies in fish proposed a core microbiota of for example 10 OTUs in Atlantic cod (Star et al., 2013), 21 OTUs in zebrafish (Roeselers et al., 2011) and 52 OTUs in rainbow trout (Wong et al., 2013). In the present study 19 OTUs were shared among all samples regardless of holding condition, albeit at different proportional abundance for each sample. Several of those OTUs reflected some of the most dominant community members like *Ruminococcaceae*, *Pseudomonas* spp. and *Mycoplasmataceae*, but rarer sequences like *Propionibacterium* spp. and *Achromobacter* spp. were also found in the core group.

In the present study we also found accessory cores, with unique core OTUs found within treatment groups – aquarium fish harboured an additional 4 OTUs, whereas loch samples had an additional 13 unique core microbiota. These results may indicate that the environment shapes aspects of the gut microbiota of Atlantic salmon parr, with a higher shared number of OTUs within the semi-natural loch environment, but also confirms maintenance of bacteria regardless of the environment and may be dictated by the gut physiology of the host. Unfortunately, at this point, too little is known about intestinal bacteria in fish to speculate on the functional relevance of the group-specific microbiota. Future metagenomics studies will be crucial to link the knowledge of what bacterial species are present to what the functional relevance of these species is.

The present study is the first in-depth analysis of gut microbiota in Atlantic salmon at the freshwater stage. By using an Illumina NGS approach we were able to detect 1850 quality-controlled OTUs. This is a marked improvement compared to the detection ability of previously used techniques, such as direct bacterial culture, DGGE, TGGE and T-RFPL, which has limited the number of bacterial species identified in the fish intestine in the past (Romero and Navarrete, 2006, Hovda et al., 2007, Navarrete et al., 2009a, Green et al., 2013). The individual fish harboured a mean of 342 OTUs. For humans it has been suggested that between 1000 and 1150 species exist overall (Lee and Mazmanian, 2010). The higher number of OTUs we identified might be due to a different OTU picking method or to a lack of information for fish specific bacteria within the microbial databases. Here we show that the gut microbiota of Atlantic salmon parr is very diverse with a handful of phyla but deep-branching richness in potential species (OTUs), which is comparable to what has been found in NGS studies in humans (Benson et al., 2010) and other fish, like rainbow trout (Ingerslev et al., 2014b).

The most dominant phylum overall was *Firmicutes*, followed by *Proteobacteria* and *Tenericutes*. However, there was a greater presence of *Tenericutes* in samples from aquarium fish whereas *Proteobacteria* were more abundant in the loch sampled fish. The dominance of these phyla is in agreement with earlier studies on Atlantic salmon and rainbow trout using both DGGE and NGS (Abid et al., 2013, Green et al., 2013, Ingerslev et al., 2014b). This differs from mammals were *Firmicutes* and *Bacteroidetes* tend to dominate (Ley et al., 2008).

*Mycoplasmataceae* (phylum *Tenericutes*) was the second most abundant family in the aquarium fish samples, and were less abundant in loch sampled fish. Metastats analysis found two OTUs associated with the family *Mycoplasmataceae* significantly more abundant in aquarium samples than loch samples (Supplementary Table S3) and one OTU of the family *Mycoplasmataceae* unique in fish from the aquarium (Supplementary Table S1). Previous studies found *Mycoplasmataceae* to be dominant in Atlantic salmon reared in seawater by DGGE and TRF and hence they appear to be a common microbiota member across environments (Abid et al., 2013, Green et al., 2013). *Mycoplasmataceae* have therefore been suggested as a dominant community member in Atlantic salmon, although the species this family comprises may be host, tissue and habitat specific (Green et al., 2013, Givens et al., 2015). In this study, OTUs mapped to genera that are known to include pathogenic species, showed a significantly higher relative abundance in loch samples compared to aquarium samples, including *Photobacterium*, *Aliivibrio*, *Aeromonas*, *Yersinia* and *Chryseobacterium* (Supplementary Table S3). Furthermore, OTUs belonging to the genera *Pseudomonas* and *Flavobacterium* were found more often at significant levels in loch samples (Supplementary Table S2). The presence of these genera shows that they can be present at low levels with no pathological effect on the fish, most likely as they are maintained at low levels by the healthy microbial community (Austin, 2006). It should be noted that sequencing short amplicons (such as Illumina) is only able to confidently assign taxonomy up to the genus level. Genera can include multiple species and strains with varying pathogenicity, for example, some non-pathogenic *Aeromonas* spp. may perform important roles in digestion by secreting several proteases (Nayak, 2010).

Lactic acid bacteria (LAB) can stimulate anti-inflammatory signals and are commonly used as probiotics to treat intestinal inflammatory disorders (Balcázar et al., 2007, Navarrete et al., 2013). We found several LAB associated OTUs that were at higher abundance in the loch fish, including 4 OTUs for *Lactobacillus*, 1 OTU for *Weissella*, and 1 OTU for *Vagococcus* (Supplementary Table S3). Furthermore we identified further OTUs mapping to the genera *Carnobacterium*, *Lactobacillus*, *Lactococcus* and *Bifidobacteria* that were only present in loch samples (Supplementary Table S2). The only LAB OTUs that were uniquely present in aquarium samples were 4 OTUs identified to the genera of *Lactobacillus* (Supplementary Table S1).

Although the NGS approach greatly improved the level of taxonomic information on the microbiota of Atlantic salmon parr, information stored in the microbial databases is not exhaustive and currently limiting microbial profiling. Not all OTUs found in this study could be confidently assigned to genus level, which is either due to lack of confidence as to where the OTU fits or it matches to a branch for which little taxonomic information is published (Goodrich et al., 2014). Members of the same genus are not necessarily closely related (Goodrich et al., 2014). Therefore, OTUs that are combined in “Other Bacteria” are an arbitrary mix (Goodrich et al., 2014). In the near future it is expected that these databases will greatly improve through the ongoing sequencing effort of bacterial genomes. Here these sequences were retained as different OTUs mapped to “Other Bacteria” were seen to be differentially present between the test groups.

To assess the diversity between the loch and aquarium groups, α-diversity metrics were calculated to identify species richness and diversity within each sample. Chao1 is a taxa-based metric used to estimate species richness in a sample and showed a significant difference between the two environmental groups, and is used extensively for intestinal microbiota studies (Chao, 1984, Hamady and Knight, 2009). Chao1 species richness was significantly greater in loch samples than aquarium samples, likely reflecting the greater diversity of bacterial species in the wild environment than in a recirculating system. A similar finding was observed in a comparative study of wild and captive killifish (*Fundulus heteroclitus*), with wild fish having higher species richness and phylogenetic diversity in gut than captive fish (Givens et al., 2015). In our data, the phylogenetic method PD_whole_tree did not reveal significant differences between loch and aquarium groups, indicating whilst there may be differences in species richness based on OTUs, when taxonomy is taken into account those differences even out as the different OTUs may be mapped to the same or similar taxonomic assignment (Hamady and Knight, 2009).

To assess β-diversity we used unweighted and weighted UniFrac (Lozupone and Knight, 2005, Lozupone et al., 2007). Whilst unweighted UniFrac detects differences in presence or absence of bacterial OTUs in different communities, the weighted UniFrac also includes the difference of abundances even when the overall groups of organisms that are present in each sample remain the same (Lozupone et al., 2007). We found that unweighted UniFrac showed clear statistically significant clustering by environmental group in both MDS plot (Fig. 4) and UPGMA tree (data not shown). This reflects the findings of the Metastats analysis showing a number of OTUs only present in either group, albeit most of these OTUs were of low relative abundance (Supplementary Tables S1, S2 and S3), and the indication of higher species richness of loch samples suggested by Chao1. However, the weighted UniFrac approach did not reveal any significant clustering. Although differences in the abundance of several taxa were observed, it is very likely that the high inter-individual variation regardless of environmental group hampered any detection of a pattern.

The detected differences in community composition between groups and individuals are most likely a reflection of the environment rather than host physiological pressures, as previously suggested (Star et al., 2013). The loch fish are likely to be continually exposed to a much greater variety of bacteria as there is a full natural community with fish also being able to eat additional food items above and beyond the commercial salmon feed presented. It is also presumed that the water itself has a greater bacterial content and diversity than in a recirculated system. All fish in the study are a farmed strain and have a similar genetic background, and since both groups were reared in a similar hatchery environment it can be expected that similar initial communities were established. Both groups of fish were fed similar diets which represent commercial salmon feeds for Atlantic salmon parr in freshwater.

However, the identification of a shared core microbiota as well as differences in core microbiota and presence/absence of certain OTUs within and between the two groups does not only indicate that habitat shapes the gut microbiota but also that there appears to be a need for colonisation by certain microbes indicating host physiological selection. Environmental rearing conditions on gut microbiota have been examined for zebrafish that indicate host selective processes play a role in the bacterial community (Roeselers et al., 2011). Zebrafish from different lab facilities and the wild shared a core of 21 OTUs, a similar observation to our data for Atlantic salmon (Roeselers et al., 2011). Furthermore several taxa were enriched in the fish gut when compared to water samples, further indicating selection mechanisms by the host (Roeselers et al., 2011).

The mechanisms and selective pressures that are most likely involved in establishment of the gut microbiota are complex (Roeselers et al., 2011). Stochastic processes may also markedly influence the inter-individual variation in a founder takes all scenario, where a random first coloniser determines the composition of later arriving species at first colonisation at hatching (Star et al., 2013). The higher bacterial species richness in the intestine of fish reared in the open loch supports this random stochasticity, whereas the identified core microbiota indicates active maintenance of crucial OTUs by the host. Further in-depth studies are needed to resolve the interplay of environmental pressures and host selective mechanisms.

In conclusion, this is the first in-depth NGS study of the intestinal microbiota of Atlantic salmon parr. The findings support previous studies, and expand on their limitations caused by technical inferior methods. The taxonomic information gathered here will be of great use to define the healthy gut microbiota and the identification of indicator species that could be used in the future to monitor the gut health status of farmed fish at this developmental stage. NGS studies investigating the microbiome of Atlantic salmon at the seawater stage have been undertaken previously (Zarkasi et al., 2014, Zarkasi et al., 2016) and are of great importance to understand and define the shifts of healthy gut microbiota throughout all life stages and thereby aquaculture production stages.

Furthermore we showed that there are significant differences between microbiota profiles of fish kept in different rearing environments, with higher species richness in fish exposed to a more natural open loch environment. Interestingly, some of those significant differences were found in genera containing potentially pathogenic species and LAB, both with higher abundances and even exclusive presence in fish from the semi-natural environment. This may lead to further investigations into a hypothetical “clean world” hypothesis applicable to fish, potentially indicating that fish in the open loch environment could be more resilient to certain pathogens by maintaining a bigger repertoire of LAB to keep them in check. Although difference in absence/presence of bacterial taxa was found significant between the groups, all fish shared a core microbiota of 19 OTUs that reflects some highly abundant taxa. These results strongly indicate that the acquisition and maintenance of gut microbiota is a complex process, which is dictated by both environmental availability of potential microbial colonisers and host physiological pressures leading to maintenance of specific bacterial taxa.

## Appendix A Supplementary data

Supplementary tables: S1 OTUs unique to the distal intestine of Atlantic salmon (*Salmo salar*) parr kept in a recirculating aquarium facility. S2 OTUs unique to the distal intestine of Atlantic salmon (*Salmo salar*) parr kept in an open loch environment. S3 OTUs found at significantly different abundance in the distal intestine of Atlantic salmon (*Salmo salar*) parr kept in a recirculating aquarium facility and the distal intestine of Atlantic salmon (*Salmo salar*) parr kept in an open loch system. Image 1
