# Supplementary material for: Environmental and physiological factors shape the gut microbiota of Atlantic salmon parr (Salmo salar L.)
Source: Aquaculture. 2017 Jan 20;467:149–57. doi: 10.1016/j.aquaculture.2016.07.017 (PMC5142738; doi:10.1016/j.aquaculture.2016.07.017)
Supplement: Supplementary file 1 — Supplementary tables: S1 OTUs unique to the distal intestine of Atlantic salmon (Salmo salar) parr kept in a recirculating aquarium facility. S2 OTUs unique to the distal intestine of Atlantic salmon (Salmo salar) parr kept in an open loch environment. S3 OTUs found at significantly different abundance in the distal intestine of Atlantic salmon (Salmo salar) parr kept in a recirculating aquarium facility and the distal intestine of Atlantic salmon (Salmo salar) parr kept in an open loch system. [file mmc1.docx]

**Supplementary table 1: Full list of Operational Taxonomic Units (OTUs) found uniquely in the distal intestine of Atlantic salmon (*Salmo salar*) parr kept in a recirculating aquarium facility, as identified by Metastats analysis.**

| **denovo^1^** | | **Greengenes ID^2^** | **Phylum** | **Genus** | **BLAST % identity ^3^** | **mean abundance (%)** | **pvalue** |
| --- | --- | --- | --- | --- | --- | --- | --- |
| 7355 | | 511685 | Acidobacteria | Other Gp1 | 94.8 | 0.1494 | 0.001 |
| 48130 | | 4367782 | Acidobacteria | Other Gp1 | 98.8 | 0.0188 | 0.001 |
| 30865 | | 4359889 | Acidobacteria | Other Gp3 | 97.8 | 0.0132 | 0.001 |
|  | |  |  |  |  |  |  |
| 35393 | | 12564 | Actinobacteria | Actinomyces | 93.8 | 0.0012 | 0.001 |
| 49322 | | 872701 | Actinobacteria | Atopobium | 96.8 | 0.0020 | 0.001 |
| 45664 | | 304693 | Actinobacteria | Cellulomonas | 96.6 | 0.0007 | 0.001 |
| 26150 | | 199919 | Actinobacteria | Conexibacter | 91.3 | 0.0005 | 0.001 |
| 50675 | | 545862 | Actinobacteria | Corynebacterium | 92.9 | 0.0015 | 0.001 |
| 49910 | | 894047 | Actinobacteria | Illumatobacter | 97.8 | 0.0010 | 0.001 |
| 9976 | | 354765 | Actinobacteria | Micromonospora | 97.3 | 0.0004 | 0.001 |
| 34434 | | 104135 | Actinobacteria | Mobiluncus | 93.8 | 0.0022 | 0.001 |
| 24490 | | 1072801 | Actinobacteria | Nakamurella | 97.3 | 0.0013 | 0.001 |
| 7044 | | 1000876 | Actinobacteria | Nocardioides | 94.1 | 0.0010 | 0.001 |
| 11650 | | 328081 | Actinobacteria | Nocardioides | 92.6 | 0.0021 | 0.001 |
| 29159 | | 12762 | Actinobacteria | Nocardioides | 97.1 | 0.0010 | 0.001 |
| 40803 | | 4339349 | Actinobacteria | Nocardioides | 94.3 | 0.0035 | 0.001 |
| 28453 | | 1145707 | Actinobacteria | Nocardiopsis | 94.3 | 0.0004 | 0.001 |
| 5013 | | 207912 | Actinobacteria | Other Actinobacteria | 95.3 | 0.0009 | 0.001 |
| 5479 | | 550302 | Actinobacteria | Other Actinobacteria | 91.3 | 0.0015 | 0.001 |
| 30409 | | 962282 | Actinobacteria | Other Actinobacteria | 92.0 | 0.0013 | 0.001 |
| 45071 | | 817152 | Actinobacteria | Other Actinobacteria | 97.3 | 0.0020 | 0.001 |
| 51587 | | 1068936 | Actinobacteria | Other Actinobacteria | 98.5 | 0.0012 | 0.001 |
| 26252 | | 624227 | Actinobacteria | Other Actinomycetales | 97.3 | 0.0012 | 0.001 |
| 26934 | | 893041 | Actinobacteria | Other Coriobacteriaceae | 96.8 | 0.0020 | 0.001 |
| 34727 | | 1145616 | Actinobacteria | Other Geodermatophilaceae | 95.6 | 0.0022 | 0.001 |
| 42909 | | 237179 | Actinobacteria | Other Intrasporangiaceae | 95.1 | 0.0007 | 0.001 |
| 40126 | | 960234 | Actinobacteria | Other Solirubrobacterales | 91.1 | 0.0032 | 0.001 |
| 10210 | | 328472 | Actinobacteria | Varibaculum | 94.7 | 0.0032 | 0.001 |
| 17671 | | 1072424 | Actinobacteria | Friedmanniella | 96.1 | 0.0004 | 0.007 |
| 6094 | | 1667535 | Actinobacteria | Ornithinimicrobium | 97.1 | 0.0004 | 0.007 |
| 46779 | | 114621 | Actinobacteria | Other Actinomycetales | 98.3 | 0.0006 | 0.007 |
| 22078 | | 13332 | Actinobacteria | Williamsia | 97.3 | 0.0007 | 0.007 |
|  | |  |  |  |  |  |  |
| 508 | | 4390319 | Bacteroidetes | Bacteroides | 93.8 | 0.0008 | 0.001 |
| 7640 | | 321972 | Bacteroidetes | Bacteroides | 87.4 | 0.0030 | 0.001 |
| 1741 | | 1002658 | Bacteroidetes | Chitinophaga | 94.1 | 0.0014 | 0.001 |
| 48143 | | 4354769 | Bacteroidetes | Flavobacterium | 93.8 | 0.0012 | 0.001 |
| 9117 | | 2312727 | Bacteroidetes | Hymenobacter | 93.6 | 0.0036 | 0.001 |
| 33465 | | 824244 | Bacteroidetes | Hymenobacter | 93.8 | 0.0008 | 0.001 |
| 35000 | | 636755 | Bacteroidetes | Hymenobacter | 94.0 | 0.0010 | 0.001 |
| 24714 | | 1124852 | Bacteroidetes | Maribacter | 93.8 | 0.0050 | 0.001 |
| 28086 | | 4397087 | Bacteroidetes | Mucilaginibacter | 93.1 | 0.0029 | 0.001 |
| 2391 | | 800713 | Bacteroidetes | Other Bacteroidetes | 91.5 | 0.0015 | 0.001 |
| 4442 | | 223126 | Bacteroidetes | Other Bacteroidetes | 92.9 | 0.0040 | 0.001 |
| 7512 | | 231330 | Bacteroidetes | Other Bacteroidetes | 92.4 | 0.0005 | 0.001 |
| 15092 | | 551126 | Bacteroidetes | Other Bacteroidetes | 94.1 | 0.0008 | 0.001 |
| 20104 | | 297337 | Bacteroidetes | Other Bacteroidetes | 91.4 | 0.0038 | 0.001 |
| 5143 | | 4203053 | Bacteroidetes | Other Chitinophagaceae | 93.4 | 0.0008 | 0.001 |
| 28473 | | 4478053 | Bacteroidetes | Other Chitinophagaceae | 91.9 | 0.0008 | 0.001 |
| 14251 | | 81782 | Bacteroidetes | Other Cryomorphaceae | 91.9 | 0.0008 | 0.001 |
| 42547 | | 702999 | Bacteroidetes | Other Cryomorphaceae | 93.1 | 0.0005 | 0.001 |
| 26768 | | 966903 | Bacteroidetes | Other Cytophagaceae | 91.5 | 0.0010 | 0.001 |
| 29178 | | 966903 | Bacteroidetes | Other Cytophagaceae | 92.7 | 0.0031 | 0.001 |
| 29311 | | 4324196 | Bacteroidetes | Other Prevotellaceae | 93.8 | 0.0026 | 0.001 |
| 13635 | | 1639372 | Bacteroidetes | Other Sphingobacteriales | 91.0 | 0.0019 | 0.001 |
| 15590 | | 231673 | Bacteroidetes | Other Sphingobacteriales | 92.2 | 0.0027 | 0.001 |
| 43814 | | 4418354 | Bacteroidetes | Planobacterium | 93.8 | 0.0017 | 0.001 |
| 9026 | | 692756 | Bacteroidetes | Porphyromonas | 94.1 | 0.0046 | 0.001 |
| 32211 | | 979707 | Bacteroidetes | Porphyromonas | 93.4 | 0.0125 | 0.001 |
| 15543 | | 173565 | Bacteroidetes | Prevotella | 94.1 | 0.0028 | 0.001 |
| 19942 | | 760967 | Bacteroidetes | Prevotella | 93.8 | 0.0066 | 0.001 |
| 20210 | | 741701 | Bacteroidetes | Prevotella | 93.8 | 0.0016 | 0.001 |
| 48927 | | 4422872 | Bacteroidetes | Sediminibacterium | 94.1 | 0.0007 | 0.001 |
| 35857 | | 4324652 | Bacteroidetes | Spirosoma | 93.4 | 0.0021 | 0.001 |
| 46475 | | 4332466 | Bacteroidetes | Spirosoma | 93.4 | 0.0045 | 0.001 |
| 3647 | | 966903 | Bacteroidetes | Hymenobacter | 92.4 | 0.0004 | 0.007 |
| 37621 | | 983599 | Bacteroidetes | Persicitalea | 92.4 | 0.0006 | 0.007 |
| 13418 | | 534367 | Bacteroidetes | Mucilaginibacter | 93.6 | 0.0003 | 0.038 |
| 39704 | | 955932 | Bacteroidetes | Other Bacteroidetes | 92.9 | 0.0002 | 0.038 |
| 42262 | | 2469654 | Bacteroidetes | Other Prevotellaceae | 93.1 | 0.0003 | 0.038 |
|  | |  |  |  |  |  |  |
| 22579 | | 249763 | Chlamydiae | Other Parachlamydiaceae | 87.2 | 0.0025 | 0.001 |
|  | |  |  |  |  |  |  |
| 14567 | | 300528 | Chloroflexi | Other Chloroflexi | 96.0 | 0.0021 | 0.001 |
|  | |  |  |  |  |  |  |
| 2038 | | 4467411 | Chloroplast | Streptophyta | 94.6 | 0.0003 | 0.003 |
|  | |  |  |  |  |  |  |
| 1368 | | 389682 | Deinococcus-Thermus | Deinococcus | 93.3 | 0.0015 | 0.001 |
| 47700 | | 2874960 | Deinococcus-Thermus | Deinococcus | 94.2 | 0.0007 | 0.001 |
|  | |  |  |  |  |  |  |
| 16381 | | 1013166 | Firmicutes | Allofustis | 91.3 | 0.0008 | 0.001 |
| 12384 | | 885628 | Firmicutes | Alloiococcus | 93.0 | 0.0036 | 0.001 |
| 41561 | | 686219 | Firmicutes | Ammoniphilus | 91.2 | 0.0016 | 0.001 |
| 17868 | | 2296122 | Firmicutes | Anaerosphaera | 98.3 | 0.0066 | 0.001 |
| 25272 | | 4330001 | Firmicutes | Anaerovorax | 91.3 | 0.0015 | 0.001 |
| 39652 | | 261139 | Firmicutes | Bacillus | 92.5 | 0.0028 | 0.001 |
| 9162 | | 4298398 | Firmicutes | Clostridium | 98.3 | 0.0010 | 0.001 |
| 10194 | | 904668 | Firmicutes | Clostridium | 97.5 | 0.0005 | 0.001 |
| 28282 | | 4430731 | Firmicutes | Erysipelothrix | 92.7 | 0.0006 | 0.001 |
| 46838 | | 4345999 | Firmicutes | Erysipelothrix | 90.4 | 0.0013 | 0.001 |
| 15240 | | 4411596 | Firmicutes | Lactobacillus | 92.0 | 0.0013 | 0.001 |
| 15280 | | 295654 | Firmicutes | Lactobacillus | 93.0 | 0.0004 | 0.001 |
| 19077 | | 131702 | Firmicutes | Lactobacillus | 92.5 | 0.0034 | 0.001 |
| 7299 | | 4376351 | Firmicutes | Other Bacillaceae | 90.0 | 0.0096 | 0.001 |
| 31034 | | 4376351 | Firmicutes | Other Bacillaceae | 89.5 | 0.0025 | 0.001 |
| 51480 | | 4441077 | Firmicutes | Other Bacillaceae | 90.2 | 0.0009 | 0.001 |
| 50216 | | 106843 | Firmicutes | Other Bacillales | 93.5 | 0.0009 | 0.001 |
| 12719 | | 829083 | Firmicutes | Other Clostridiales | 97.3 | 0.0006 | 0.001 |
| 14423 | | 755148 | Firmicutes | Other Clostridiales | 98.8 | 0.0054 | 0.001 |
| 16438 | | 320087 | Firmicutes | Other Clostridiales | 98.8 | 0.0004 | 0.001 |
| 27760 | | 3805726 | Firmicutes | Other Clostridiales | 97.3 | 0.0040 | 0.001 |
| 37375 | | 523577 | Firmicutes | Other Incertae_Sedis_XI | 98.3 | 0.0100 | 0.001 |
| 38691 | | 495052 | Firmicutes | Other Incertae_Sedis_XI | 97.8 | 0.0030 | 0.001 |
| 44943 | | 963606 | Firmicutes | Other Incertae_Sedis_XI | 98.8 | 0.0083 | 0.001 |
| 48283 | | 841344 | Firmicutes | Other Incertae_Sedis_XI | 98.8 | 0.0055 | 0.001 |
| 8280 | | 175751 | Firmicutes | Other Lachnospiraceae | 98.5 | 0.0019 | 0.001 |
| 11712 | | 176332 | Firmicutes | Other Lachnospiraceae | 98.5 | 0.0077 | 0.001 |
| 20383 | | 296872 | Firmicutes | Other Lachnospiraceae | 97.0 | 0.0018 | 0.001 |
| 13422 | | 494906 | Firmicutes | Peptoniphilus | 98.8 | 0.0017 | 0.001 |
| 30514 | | 4400372 | Firmicutes | Thermoactinomyces | 91.6 | 0.0003 | 0.003 |
| 47774 | | 137043 | Firmicutes | Lactobacillus | 89.4 | 0.0003 | 0.007 |
| 9919 | | 54730 | Firmicutes | Other Erysipelotrichaceae | 89.3 | 0.0003 | 0.007 |
| 3245 | | 181589 | Firmicutes | Staphylococcus | 91.1 | 0.0013 | 0.007 |
| 3778 | | 188659 | Firmicutes | Blautia | 98.3 | 0.0018 | 0.017 |
| 37654 | | 653394 | Firmicutes | Brevibacillus | 92.3 | 0.0005 | 0.017 |
| 17623 | | 196713 | Firmicutes | Coprococcus | 98.5 | 0.0003 | 0.017 |
| 26291 | | 676447 | Firmicutes | Other Bacillaceae | 90.7 | 0.0002 | 0.017 |
| 36276 | | 365621 | Firmicutes | Other Lachnospiraceae | 98.8 | 0.0002 | 0.017 |
| 13650 | | 561607 | Firmicutes | Other Ruminococcaceae | 88.8 | 0.0003 | 0.017 |
| 22687 | | 98605 | Firmicutes | Streptococcus | 92.3 | 0.0003 | 0.017 |
| 28472 | | 348009 | Firmicutes | Oscillibacter | 98.0 | 0.0003 | 0.038 |
| 13714 | | 2207032 | Firmicutes | Other Clostridiales | 96.3 | 0.0004 | 0.038 |
| 24565 | | 389529 | Firmicutes | Other Clostridiales | 85.0 | 0.0002 | 0.038 |
| 23140 | | 4466275 | Firmicutes | Other Ruminococcaceae | 86.5 | 0.0003 | 0.038 |
|  | |  |  |  |  |  |  |
| 25072 | | 16854 | OD1 | Other OD1_genera_incertae_sedis | 86.0 | 0.0016 | 0.001 |
| 51047 | | 106562 | OD1 | Other OD1_genera_incertae_sedis | 94.6 | 0.0042 | 0.001 |
| 660 | | 254787 | OD1 | Other OD1_genera_incertae_sedis | 89.9 | 0.0014 | 0.001 |
| 4433 | | 531358 | OD1 | Other OD1_genera_incertae_sedis | 94.3 | 0.0005 | 0.001 |
| 51427 | | 650048 | OD1 | Other OD1_genera_incertae_sedis | 91.9 | 0.0004 | 0.001 |
| 40836 | | 773120 | OD1 | Other OD1_genera_incertae_sedis | 93.8 | 0.0011 | 0.001 |
| 39343 | | 1132720 | OD1 | Other OD1_genera_incertae_sedis | 91.4 | 0.0014 | 0.001 |
| 16098 | | 3123102 | OD1 | Other OD1_genera_incertae_sedis | 83.5 | 0.0062 | 0.001 |
| 37702 | | 3123102 | OD1 | Other OD1_genera_incertae_sedis | 84.5 | 0.0004 | 0.001 |
| 622 | | 4322208 | OD1 | Other OD1_genera_incertae_sedis | 87.0 | 0.0020 | 0.001 |
| 37158 | | 4322208 | OD1 | Other OD1_genera_incertae_sedis | 85.4 | 0.0012 | 0.001 |
| 4360 | | 4396699 | OD1 | Other OD1_genera_incertae_sedis | 85.2 | 0.0033 | 0.001 |
| 33349 | | 4439807 | OD1 | Other OD1_genera_incertae_sedis | 91.1 | 0.0004 | 0.001 |
| 45045 | | 4443105 | OD1 | Other OD1_genera_incertae_sedis | 86.0 | 0.0020 | 0.001 |
| 21339 | | 4467411 | OD1 | Other OD1_genera_incertae_sedis | 91.2 | 0.0004 | 0.001 |
| 50414 | | 4467411 | OD1 | Other OD1_genera_incertae_sedis | 92.4 | 0.0008 | 0.001 |
| 38473 | | 4477111 | OD1 | Other OD1_genera_incertae_sedis | 88.7 | 0.0007 | 0.001 |
| 4651 | | 105625 | OD1 | Other OD1_genera_incertae_sedis | 83.7 | 0.0003 | 0.003 |
| 33467 | | 551628 | OD1 | Other OD1_genera_incertae_sedis | 87.6 | 0.0006 | 0.003 |
| 44342 | | 4467411 | OD1 | Other OD1_genera_incertae_sedis | 88.0 | 0.0006 | 0.003 |
| 42049 | | 4322208 | OD1 | Other OD1_genera_incertae_sedis | 88.1 | 0.0003 | 0.007 |
| 45215 | | 4375615 | OD1 | Other OD1_genera_incertae_sedis | 86.7 | 0.0004 | 0.007 |
| 8092 | | 4443105 | OD1 | Other OD1_genera_incertae_sedis | 90.1 | 0.0002 | 0.017 |
| 18642 | | 678059 | OD1 | Other OD1_genera_incertae_sedis | 88.0 | 0.0002 | 0.038 |
| 18499 | | 4443105 | OD1 | Other OD1_genera_incertae_sedis | 94.3 | 0.0002 | 0.038 |
|  | |  |  |  |  |  |  |
| 15757 | | 359924 | Planctomycetes | Gemmata | 95.6 | 0.0018 | 0.001 |
| 23673 | | 4075738 | Planctomycetes | Other Plantomycetaceae | 91.7 | 0.0007 | 0.001 |
| 34181 | | 416617 | Planctomycetes | Other Planctomycetaceae | 93.9 | 0.0023 | 0.001 |
| 42048 | | 551223 | Planctomycetes | Planctomyces | 94.4 | 0.0008 | 0.001 |
|  | |  |  |  |  |  |  |
| 34131 | | 248521 | Proteobacteria | Acinetobacter | 92.5 | 0.0099 | 0.001 |
| 42129 | | 106476 | Proteobacteria | Aliivibrio | 89.9 | 0.0015 | 0.001 |
| 41224 | | 892591 | Proteobacteria | Bdellovibrio | 94.8 | 0.0009 | 0.001 |
| 26707 | | 1908796 | Proteobacteria | Caldimonas | 92.3 | 0.0031 | 0.001 |
| 24838 | | 749805 | Proteobacteria | Cupriavidus | 84.1 | 0.0112 | 0.001 |
| 45139 | | 3437276 | Proteobacteria | Cupriavidus | 92.5 | 0.0008 | 0.001 |
| 35763 | | 329118 | Proteobacteria | Halomonas | 92.7 | 0.0014 | 0.001 |
| 2103 | | 4431353 | Proteobacteria | Legionella | 91.6 | 0.0006 | 0.001 |
| 34221 | | 4477963 | Proteobacteria | Legionella | 90.2 | 0.0034 | 0.001 |
| 42382 | | 833330 | Proteobacteria | Lysobacter | 82.9 | 0.0006 | 0.001 |
| 27881 | | 620677 | Proteobacteria | Massilia | 92.7 | 0.0044 | 0.001 |
| 3457 | | 1015007 | Proteobacteria | Methyloversatilis | 92.3 | 0.0063 | 0.001 |
| 2934 | | 516198 | Proteobacteria | Neisseria | 92.5 | 0.0024 | 0.001 |
| 31545 | | 1106060 | Proteobacteria | Neisseria | 82.9 | 0.0009 | 0.001 |
| 19607 | | 364038 | Proteobacteria | Other Acetobacteriaceae | 94.0 | 0.0012 | 0.001 |
| 32884 | | 4464578 | Proteobacteria | Other Alphaproteobacteria | 85.6 | 0.0018 | 0.001 |
| 5392 | | 570344 | Proteobacteria | Other Alphaproteobacteria | 98.5 | 0.0071 | 0.001 |
| 43375 | | 153512 | Proteobacteria | Other Betaproteobacteria | 91.6 | 0.0008 | 0.001 |
| 37346 | | 773739 | Proteobacteria | Other Comamonadaceae | 92.5 | 0.0062 | 0.001 |
| 38927 | | 3581985 | Proteobacteria | Other Comamonadaceae | 92.5 | 0.0020 | 0.001 |
| 50256 | | 278887 | Proteobacteria | Other Comamonadaceae | 92.3 | 0.0006 | 0.001 |
| 44441 | | 584900 | Proteobacteria | Other Erythrobacteriaceae | 98.5 | 0.0012 | 0.001 |
| 3773 | | 4342460 | Proteobacteria | Other Gammaproteobacteria | 89.9 | 0.0029 | 0.001 |
| 23951 | | 846474 | Proteobacteria | Other Gammaproteobacteria | 90.9 | 0.0013 | 0.001 |
| 39936 | | 1127760 | Proteobacteria | Other Gammaproteobacteria | 93.0 | 0.0007 | 0.001 |
| 40731 | | 213493 | Proteobacteria | Other Gammaproteobacteria | 91.8 | 0.0012 | 0.001 |
| 45394 | | 666738 | Proteobacteria | Other Gammaproteobacteria | 89.9 | 0.0017 | 0.001 |
| 38306 | | 4482366 | Proteobacteria | Other Legionellaceae | 90.4 | 0.0021 | 0.001 |
| 49921 | | 2669168 | Proteobacteria | Other Nannocystaceae | 91.7 | 0.0008 | 0.001 |
| 43868 | | 900973 | Proteobacteria | Other Neisseriaceae | 93.0 | 0.0021 | 0.001 |
| 21591 | | 499916 | Proteobacteria | Other Oceanospirillales | 87.8 | 0.0008 | 0.001 |
| 1656 | | 4404862 | Proteobacteria | Other Proteobacteria | 95.8 | 0.0020 | 0.001 |
| 25317 | | 1053100 | Proteobacteria | Other Proteobacteria | 97.3 | 0.0018 | 0.001 |
| 38188 | | 233724 | Proteobacteria | Other Proteobacteria | 98.8 | 0.0006 | 0.001 |
| 45956 | | 787865 | Proteobacteria | Other Proteobacteria | 91.1 | 0.0007 | 0.001 |
| 37859 | | 906396 | Proteobacteria | Other Rhizobiales | 98.0 | 0.0009 | 0.001 |
| 48560 | | 531803 | Proteobacteria | Other Rhizobiales | 97.5 | 0.0040 | 0.001 |
| 24974 | | 100870 | Proteobacteria | Other Rhodobacteraceae | 97.0 | 0.0012 | 0.001 |
| 42502 | | 105849 | Proteobacteria | Other Rhodospirillales | 96.3 | 0.0018 | 0.001 |
| 44945 | | 141461 | Proteobacteria | Other Xanthomonadaceae | 92.5 | 0.0021 | 0.001 |
| 45095 | | 2628214 | Proteobacteria | Pedomicrobium | 94.3 | 0.0013 | 0.001 |
| 30950 | | 239066 | Proteobacteria | Peredibacter | 91.6 | 0.0025 | 0.001 |
| 13302 | | 591923 | Proteobacteria | Pseudoalteromonas | 93.0 | 0.0028 | 0.001 |
| 27631 | | 1566691 | Proteobacteria | Pseudomonas | 90.4 | 0.0008 | 0.001 |
| 35711 | | 4349788 | Proteobacteria | Pseudomonas | 92.0 | 0.0008 | 0.001 |
| 3453 | | 4345543 | Proteobacteria | Pseudoxanthomonas | 92.7 | 0.0031 | 0.001 |
| 20442 | | 1105919 | Proteobacteria | Ralstonia | 91.6 | 0.0054 | 0.001 |
| 12947 | | 4479555 | Proteobacteria | Roseomonas | 97.8 | 0.0040 | 0.001 |
| 47591 | | 365967 | Proteobacteria | Rubellimicrobium | 98.3 | 0.0012 | 0.001 |
| 42658 | | 544841 | Proteobacteria | Sphingomonas | 98.5 | 0.0024 | 0.001 |
| 48114 | | 1111944 | Proteobacteria | Sphingomonas | 97.0 | 0.0014 | 0.001 |
| 17784 | | 636296 | Proteobacteria | Sutterella | 90.4 | 0.0006 | 0.001 |
| 33213 | | 972024 | Proteobacteria | Bdellovibrio | 96.5 | 0.0003 | 0.003 |
| 12249 | | 4347970 | Proteobacteria | Kofleria | 91.5 | 0.0006 | 0.003 |
| 40812 | | 4348984 | Proteobacteria | Luteimonas | 92.0 | 0.0006 | 0.003 |
| 47336 | | 590825 | Proteobacteria | Marinomonas | 92.3 | 0.0006 | 0.003 |
| 30312 | | 820291 | Proteobacteria | Other Betaproteobacteria | 91.8 | 0.0005 | 0.003 |
| 13299 | | 3514716 | Proteobacteria | Other Burkholderiales | 91.3 | 0.0006 | 0.003 |
| 33378 | | 4375000 | Proteobacteria | Escherichia/Shigella | 82.2 | 0.0006 | 0.007 |
| 18873 | | 101884 | Proteobacteria | Other Betaproteobacteria | 92.7 | 0.0005 | 0.007 |
| 47510 | | 1116070 | Proteobacteria | Other Gammaproteobacteria | 92.7 | 0.0006 | 0.007 |
| 21240 | | 153559 | Proteobacteria | Other Rhodocyclaceae | 92.0 | 0.0003 | 0.007 |
| 22301 | | 161024 | Proteobacteria | Photobacterium | 92.7 | 0.0003 | 0.007 |
| 11510 | | 4308264 | Proteobacteria | Bdellovibrio | 97.0 | 0.0002 | 0.017 |
| 44085 | | 929312 | Proteobacteria | Roseomonas | 96.8 | 0.0005 | 0.017 |
| 14105 | | 823479 | Proteobacteria | Duganella | 83.6 | 0.0002 | 0.038 |
| 13918 | | 4457268 | Proteobacteria | Escherichia/Shigella | 90.2 | 0.0003 | 0.038 |
| 39817 | | 548253 | Proteobacteria | Other Brucellaceae | 98.3 | 0.0004 | 0.038 |
| 6875 | | 4321229 | Proteobacteria | Other Comamonadaceae | 90.6 | 0.0003 | 0.038 |
| 7646 | | 836096 | Proteobacteria | Other Gammaproteobacteria | 85.1 | 0.0002 | 0.038 |
| 30070 | | 4399988 | Proteobacteria | Pantoea | 92.3 | 0.0004 | 0.038 |
|  | |  |  |  |  |  |  |
| 5449 | | 4447514 | Spirochaetes | Treponema | 92.0 | 0.0072 | 0.001 |
|  | |  |  |  |  |  |  |
| 25871 | | 346610 | SR1 | Other SR1_genera_incertae_sedis | 95.0 | 0.0196 | 0.001 |
|  | |  |  |  |  |  |  |
| 11223 | | 818711 | Tenericutes | Mycoplasma | 86.4 | 0.0038 | 0.001 |
| 29770 | | 147311 | Tenericutes | Other Mycoplasmataceae | 87.9 | 0.0003 | 0.038 |
|  | |  |  |  |  |  |  |
| 31303 | | 37526 | TM7 | Other TM7_genera_incertae_sedis | 89.1 | 0.0006 | 0.001 |
| 24381 | | 189880 | TM7 | Other TM7_genera_incertae_sedis | 94.3 | 0.0019 | 0.001 |
| 16087 | | 222592 | TM7 | Other TM7_genera_incertae_sedis | 90.1 | 0.0056 | 0.001 |
| 44743 | | 549319 | TM7 | Other TM7_genera_incertae_sedis | 93.8 | 0.0019 | 0.001 |
| 45037 | | 940666 | TM7 | Other TM7_genera_incertae_sedis | 95.5 | 0.0027 | 0.001 |
| 49208 | | 1129474 | TM7 | Other TM7_genera_incertae_sedis | 95.3 | 0.0007 | 0.001 |
| 39808 | | 4347993 | TM7 | Other TM7_genera_incertae_sedis | 90.8 | 0.0010 | 0.001 |
| 40333 | | 834742 | TM7 | Other TM7_genera_incertae_sedis | 96.8 | 0.0003 | 0.003 |
| 8849 | | 585984 | TM7 | Other TM7_genera_incertae_sedis | 96.0 | 0.0003 | 0.007 |
| 15642 | | 21860 | TM7 | Other TM7_genera_incertae_sedis | 91.8 | 0.0002 | 0.038 |
| 1099 | | 195489 | TM7 | Other TM7_genera_incertae_sedis | 94.6 | 0.0002 | 0.038 |
|  | |  |  |  |  |  |  |
| 41632 | | 74066 | Unclassified Bacteria | Unclassified Bacteria | 83.1 | 0.0006 | 0.001 |
| 11252 | | 98258 | Unclassified Bacteria | Unclassified Bacteria | 97.8 | 0.0034 | 0.001 |
| 5840 | | 145173 | Unclassified Bacteria | Unclassified Bacteria | 80.4 | 0.0004 | 0.001 |
| 43712 | | 147311 | Unclassified Bacteria | Unclassified Bacteria | 87.4 | 0.0024 | 0.001 |
| 25598 | | 156900 | Unclassified Bacteria | Unclassified Bacteria | 92.8 | 0.0034 | 0.001 |
| 2204 | | 175067 | Unclassified Bacteria | Unclassified Bacteria | 90.9 | 0.0034 | 0.001 |
| 14220 | | 200608 | Unclassified Bacteria | Unclassified Bacteria | 97.5 | 0.0014 | 0.001 |
| 39214 | | 202110 | Unclassified Bacteria | Unclassified Bacteria | 80.2 | 0.0016 | 0.001 |
| 19063 | | 202528 | Unclassified Bacteria | Unclassified Bacteria | 81.3 | 0.0037 | 0.001 |
| 3400 | | 204757 | Unclassified Bacteria | Unclassified Bacteria | 96.3 | 0.0011 | 0.001 |
| 46324 | | 205209 | Unclassified Bacteria | Unclassified Bacteria | 93.6 | 0.0011 | 0.001 |
| 25110 | | 207510 | Unclassified Bacteria | Unclassified Bacteria | 80.8 | 0.0012 | 0.001 |
| 858 | 278587 | Unclassified Bacteria | Unclassified Bacteria | 80.6 | 0.0004 | 0.001 |  |
| 33674 | 278587 | Unclassified Bacteria | Unclassified Bacteria | 83.7 | 0.0008 | 0.001 |  |
| 37287 | 318860 | Unclassified Bacteria | Unclassified Bacteria | 92.8 | 0.0009 | 0.001 |  |
| 15780 | 335018 | Unclassified Bacteria | Unclassified Bacteria | 83.1 | 0.0039 | 0.001 |  |
| 44363 | 508877 | Unclassified Bacteria | Unclassified Bacteria | 91.1 | 0.0055 | 0.001 |  |
| 10193 | 540167 | Unclassified Bacteria | Unclassified Bacteria | 89.9 | 0.0019 | 0.001 |  |
| 36215 | 685954 | Unclassified Bacteria | Unclassified Bacteria | 94.6 | 0.0004 | 0.001 |  |
| 14279 | 807337 | Unclassified Bacteria | Unclassified Bacteria | 80.2 | 0.0007 | 0.001 |  |
| 28176 | 817141 | Unclassified Bacteria | Unclassified Bacteria | 96.0 | 0.0013 | 0.001 |  |
| 18881 | 821884 | Unclassified Bacteria | Unclassified Bacteria | 88.5 | 0.0010 | 0.001 |  |
| 20695 | 1104864 | Unclassified Bacteria | Unclassified Bacteria | 95.5 | 0.0018 | 0.001 |  |
| 35762 | 1109010 | Unclassified Bacteria | Unclassified Bacteria | 87.6 | 0.0038 | 0.001 |  |
| 8622 | 1110613 | Unclassified Bacteria | Unclassified Bacteria | 94.3 | 0.0060 | 0.001 |  |
| 37073 | 1118192 | Unclassified Bacteria | Unclassified Bacteria | 92.6 | 0.0027 | 0.001 |  |
| 4447 | 1119728 | Unclassified Bacteria | Unclassified Bacteria | 85.9 | 0.0025 | 0.001 |  |
| 19360 | 1573550 | Unclassified Bacteria | Unclassified Bacteria | 92.1 | 0.0054 | 0.001 |  |
| 49440 | 1887414 | Unclassified Bacteria | Unclassified Bacteria | 98.3 | 0.0073 | 0.001 |  |
| 28734 | 2653243 | Unclassified Bacteria | Unclassified Bacteria | 89.6 | 0.0033 | 0.001 |  |
| 6596 | 3746871 | Unclassified Bacteria | Unclassified Bacteria | 93.6 | 0.0023 | 0.001 |  |
| 6688 | 3746871 | Unclassified Bacteria | Unclassified Bacteria | 91.1 | 0.0019 | 0.001 |  |
| 20266 | 3746871 | Unclassified Bacteria | Unclassified Bacteria | 93.5 | 0.0009 | 0.001 |  |
| 51058 | 3746871 | Unclassified Bacteria | Unclassified Bacteria | 92.6 | 0.0005 | 0.001 |  |
| 40580 | 3746876 | Unclassified Bacteria | Unclassified Bacteria | 95.3 | 0.0014 | 0.001 |  |
| 13266 | 3825731 | Unclassified Bacteria | Unclassified Bacteria | 88.6 | 0.0010 | 0.001 |  |
| 38996 | 3886267 | Unclassified Bacteria | Unclassified Bacteria | 91.5 | 0.0005 | 0.001 |  |
| 3733 | 4298846 | Unclassified Bacteria | Unclassified Bacteria | 88.5 | 0.0008 | 0.001 |  |
| 12069 | 4309998 | Unclassified Bacteria | Unclassified Bacteria | 85.4 | 0.0008 | 0.001 |  |
| 2446 | 4322208 | Unclassified Bacteria | Unclassified Bacteria | 79.8 | 0.0012 | 0.001 |  |
| 8332 | 4396488 | Unclassified Bacteria | Unclassified Bacteria | 85.7 | 0.0022 | 0.001 |  |
| 3598 | 4419974 | Unclassified Bacteria | Unclassified Bacteria | 89.5 | 0.0007 | 0.001 |  |
| 22229 | 4464974 | Unclassified Bacteria | Unclassified Bacteria | 90.9 | 0.0012 | 0.001 |  |
| 21989 | 4479103 | Unclassified Bacteria | Unclassified Bacteria | 88.5 | 0.0008 | 0.001 |  |
| 12920 | 145173 | Unclassified Bacteria | Unclassified Bacteria | 87.1 | 0.0003 | 0.007 |  |
| 24072 | 3225199 | Unclassified Bacteria | Unclassified Bacteria | 95.3 | 0.0004 | 0.007 |  |
| 21846 | 4464974 | Unclassified Bacteria | Unclassified Bacteria | 89.7 | 0.0003 | 0.007 |  |
| 16485 | 335018 | Unclassified Bacteria | Unclassified Bacteria | 82.9 | 0.0002 | 0.017 |  |
| 12143 | 1892252 | Unclassified Bacteria | Unclassified Bacteria | 94.1 | 0.0002 | 0.017 |  |
| 20925 | 3746871 | Unclassified Bacteria | Unclassified Bacteria | 93.3 | 0.0003 | 0.017 |  |
| 31354 | 3746871 | Unclassified Bacteria | Unclassified Bacteria | 93.4 | 0.0003 | 0.017 |  |
| 43100 | 4425145 | Unclassified Bacteria | Unclassified Bacteria | 90.9 | 0.0002 | 0.017 |  |
| 47672 | 4464974 | Unclassified Bacteria | Unclassified Bacteria | 88.4 | 0.0018 | 0.017 |  |
| 805 | 735769 | Unclassified Bacteria | Unclassified Bacteria | 85.4 | 0.0002 | 0.038 |  |
| 18373 | 803637 | Unclassified Bacteria | Unclassified Bacteria | 91.4 | 0.0002 | 0.038 |  |
| 49160 | 2927689 | Unclassified Bacteria | Unclassified Bacteria | 93.8 | 0.0003 | 0.038 |  |
| 20448 | 4299277 | Unclassified Bacteria | Unclassified Bacteria | 92.9 | 0.0002 | 0.038 |  |
|  |  |  |  |  |  |  |  |
| 20481 | na | Unclassified Bacteria | Unclassified Bacteria | na | 0.0044 | 0.001 |  |
| 21299 | na | Unclassified Bacteria | Unclassified Bacteria | na | 0.0020 | 0.001 |  |
| 36433 | na | Unclassified Bacteria | Unclassified Bacteria | na | 0.0005 | 0.001 |  |
| 6752 | na | Unclassified Bacteria | Unclassified Bacteria | na | 0.0025 | 0.003 |  |
| 42169 | na | Unclassified Bacteria | Unclassified Bacteria | na | 0.0025 | 0.003 |  |
| 11253 | na | Unclassified Bacteria | Unclassified Bacteria | na | 0.0022 | 0.007 |  |
| 23779 | na | Unclassified Bacteria | Unclassified Bacteria | na | 0.0007 | 0.007 |  |
| 14374 | na | Unclassified Bacteria | Unclassified Bacteria | na | 0.0003 | 0.038 |  |
| 24730 | na | Unclassified Bacteria | Unclassified Bacteria | na | 0.0015 | 0.038 |  |
| 34389 | na | Unclassified Bacteria | Unclassified Bacteria | na | 0.0015 | 0.038 |  |
| 44512 | na | Unclassified Bacteria | Unclassified Bacteria | na | 0.0015 | 0.038 |  |
|  |  |  |  |  |  |  |  |
| 12365 | na | Unclassified Sequence | Unclassified Sequence | na | 0.0006 | 0.007 |  |
| 39566 | na | Unclassified Sequence | Unclassified Sequence | na | 0.0022 | 0.007 |  |
| 46589 | na | Unclassified Sequence | Unclassified Sequence | na | 0.0033 | 0.001 |  |
|  |  |  |  |  |  |  |  |
| 21045 | 592129 | Verrucomicrobia | Opitutus | 92.5 | 0.0087 | 0.001 |  |
| 40814 | 591617 | Verrucomicrobia | Verrucomicrobium | 93.5 | 0.0003 | 0.017 |  |

^1^ unique number assigned to sequences with less than 97% similarity to other sequences

^2^ number that is associated with the sequence in the aligned Greengenes database that fits the query sequence

^3^ percent identity of the query sequence to the assigned Greengenes reference sequence

**Supplementary table 2: Full list of Operational Taxonomic Units (OTUs) found uniquely in the distal intestine of Atlantic salmon (*Salmo salar*) parr kept in an open loch environment, as identified by Metastats analysis.**

| **denovo^1^** | **Greengenes ID^2^** | **Phylum** | **Genus** | **BLAST % identity ^3^** | **mean abundance (%)** | **pvalue** |
| --- | --- | --- | --- | --- | --- | --- |
| 37674 | 103580 | Acidobacteria | Other Gp3 | 94.5 | 0.0008 | 0.001 |
| 48942 | 560359 | Acidobacteria | Other Gp3 | 98.8 | 0.0035 | 0.001 |
| 39698 | 636687 | Acidobacteria | Other Gp4 | 98.0 | 0.0111 | 0.001 |
| 33166 | 806689 | Acidobacteria | Other Gp6 | 92.5 | 0.0021 | 0.001 |
| 39175 | 3270462 | Acidobacteria | Other Gp6 | 91.8 | 0.0007 | 0.002 |
|  |  |  |  |  |  |  |
| 39094 | 1146291 | Actinobacteria | Corynebacterium | 96.1 | 0.0012 | 0.001 |
| 5718 | 584954 | Actinobacteria | Olsenella | 95.6 | 0.0011 | 0.001 |
| 46883 | 1667532 | Actinobacteria | Ornithinimicrobium | 97.1 | 0.0014 | 0.001 |
| 32298 | 247757 | Actinobacteria | Other Coriobacteriaceae | 90.0 | 0.0021 | 0.001 |
| 26003 | 2232355 | Actinobacteria | Paraeggerthella | 96.0 | 0.0023 | 0.001 |
| 40341 | 244494 | Actinobacteria | Pseudonocardia | 97.3 | 0.0023 | 0.001 |
| 44037 | 253403 | Actinobacteria | Saccharopolyspora | 95.2 | 0.0027 | 0.001 |
| 6405 | 768898 | Actinobacteria | Thermobifido | 94.1 | 0.0010 | 0.002 |
| 24140 | 104963 | Actinobacteria | Other Actinomycetales | 97.5 | 0.0009 | 0.003 |
| 42990 | 173906 | Actinobacteria | Curtobacterium | 95.4 | 0.0013 | 0.006 |
| 5615 | 1047041 | Actinobacteria | Other Actinomycetales | 97.5 | 0.0087 | 0.009 |
| 14490 | 825808 | Actinobacteria | Bifidobacterium | 97.1 | 0.0006 | 0.011 |
| 34038 | 811000 | Actinobacteria | Illumatobacter | 97.3 | 0.0004 | 0.020 |
| 4083 | 811159 | Actinobacteria | Luteococcus | 97.5 | 0.0004 | 0.020 |
| 24013 | 710414 | Actinobacteria | Other Solirubrobacterales | 89.2 | 0.0009 | 0.038 |
| 23747 | 12938 | Actinobacteria | Saccharopolyspora | 96.6 | 0.0004 | 0.038 |
| 26294 | 263212 | Actinobacteria | Micrococcaceae | 95.8 | 0.0019 | 0.044 |
|  |  |  |  |  |  |  |
| 3234 | 4443200 | Bacteroidetes | Capnocytophaga | 93.8 | 0.0020 | 0.001 |
| 22168 | 752012 | Bacteroidetes | Dysgonomonas | 93.8 | 0.0023 | 0.001 |
| 18925 | 801568 | Bacteroidetes | Flavobacterium | 93.4 | 0.0011 | 0.001 |
| 24755 | 1021754 | Bacteroidetes | Flavobacterium | 92.4 | 0.0082 | 0.001 |
| 50509 | 149844 | Bacteroidetes | Marinifilum | 93.4 | 0.0007 | 0.001 |
| 10125 | 847427 | Bacteroidetes | Odoribacter | 85.3 | 0.0019 | 0.001 |
| 7167 | 159560 | Bacteroidetes | Other Bacteroidetes | 88.6 | 0.0012 | 0.001 |
| 7671 | 16733 | Bacteroidetes | Other Bacteroidetes | 93.3 | 0.0047 | 0.001 |
| 8925 | 242303 | Bacteroidetes | Other Bacteroidetes | 88.4 | 0.0043 | 0.001 |
| 14867 | 252788 | Bacteroidetes | Other Bacteroidetes | 92.9 | 0.0008 | 0.001 |
| 37960 | 361111 | Bacteroidetes | Other Bacteroidetes | 91.7 | 0.0020 | 0.001 |
| 42146 | 4469493 | Bacteroidetes | Other Bacteroidetes | 93.4 | 0.0026 | 0.001 |
| 42755 | 159560 | Bacteroidetes | Other Bacteroidetes | 87.0 | 0.0020 | 0.001 |
| 39028 | 1126475 | Bacteroidetes | Other Cytophagaceae | 84.6 | 0.0010 | 0.001 |
| 13588 | 240678 | Bacteroidetes | Other Flavobacteriaceae | 90.8 | 0.0015 | 0.001 |
| 36281 | 4433676 | Bacteroidetes | Other Flavobacteriaceae | 89.8 | 0.0009 | 0.001 |
| 33822 | 4446973 | Bacteroidetes | Other Porphyromonadaceae | 93.1 | 0.0020 | 0.001 |
| 43496 | 944401 | Bacteroidetes | Pedobacter | 93.8 | 0.0047 | 0.001 |
| 28414 | 4359222 | Bacteroidetes | Prevotella | 93.8 | 0.0014 | 0.001 |
| 28764 | 2714267 | Bacteroidetes | Prevotella | 92.9 | 0.0022 | 0.001 |
| 30546 | 705241 | Bacteroidetes | Prevotella | 93.6 | 0.0022 | 0.001 |
| 33883 | 692902 | Bacteroidetes | Prevotella | 91.0 | 0.0005 | 0.001 |
| 36223 | 4302571 | Bacteroidetes | Prevotella | 93.8 | 0.0009 | 0.001 |
| 50007 | 4331006 | Bacteroidetes | Prevotella | 93.8 | 0.0037 | 0.001 |
| 49806 | 824606 | Bacteroidetes | Sphingobacterium | 94.1 | 0.0030 | 0.001 |
| 5027 | 235391 | Bacteroidetes | Salinimicrobium | 92.4 | 0.0009 | 0.003 |
| 49278 | 808071 | Bacteroidetes | Other Chitinophagaceae | 93.3 | 0.0007 | 0.006 |
| 32025 | 4305837 | Bacteroidetes | Chryseobacterium | 93.6 | 0.0004 | 0.011 |
| 26085 | 913875 | Bacteroidetes | Flavobacterium | 92.7 | 0.0005 | 0.020 |
| 36648 | 1138568 | Bacteroidetes | Flavobacterium | 94.1 | 0.0004 | 0.020 |
| 27519 | 4310398 | Bacteroidetes | Prevotella | 91.9 | 0.0005 | 0.020 |
| 41671 | 4406621 | Bacteroidetes | Tannerella | 94.1 | 0.0003 | 0.020 |
| 11281 | 91024 | Bacteroidetes | Chryseobacterium | 92.9 | 0.0006 | 0.038 |
| 25207 | 4479603 | Bacteroidetes | Prevotella | 93.6 | 0.0009 | 0.038 |
| 30653 | 529349 | Bacteroidetes | Prevotella | 93.6 | 0.0011 | 0.039 |
|  |  |  |  |  |  |  |
| 40546 | 4326875 | Chlamydiae | Neochlamydia | 81.0 | 0.0051 | 0.001 |
| 49459 | 2930 | Chlamydiae | Neochlamydia | 86.4 | 0.0012 | 0.001 |
| 26717 | 823226 | Chlamydiae | Other Chlamydiales | 87.6 | 0.0021 | 0.001 |
| 33602 | 552935 | Chlamydiae | Other Chlamydiales | 89.7 | 0.0009 | 0.001 |
| 8590 | 552935 | Chlamydiae | Other Chlamydiales | 87.1 | 0.0007 | 0.011 |
|  |  |  |  |  |  |  |
| 47521 | 567282 | Deinococcus-Thermus | Deinococcus | 92.6 | 0.0014 | 0.001 |
|  |  |  |  |  |  |  |
| 9968 | 1142965 | Firmicutes | Acholeplasma | 96.4 | 0.0016 | 0.001 |
| 23288 | 236650 | Firmicutes | Anaerococcus | 98.5 | 0.0037 | 0.001 |
| 38493 | 3350673 | Firmicutes | Anoxybacillus | 93.0 | 0.0017 | 0.001 |
| 38974 | 159299 | Firmicutes | Bacillus | 91.4 | 0.0052 | 0.001 |
| 39471 | 325946 | Firmicutes | Bacillus | 92.0 | 0.0039 | 0.001 |
| 40541 | 183571 | Firmicutes | Bacillus | 92.3 | 0.0663 | 0.001 |
| 29432 | 4404405 | Firmicutes | Desemzia | 93.0 | 0.0015 | 0.001 |
| 37134 | 1111582 | Firmicutes | Enterococcus | 93.0 | 0.0034 | 0.001 |
| 5390 | 4331613 | Firmicutes | Gracilibacillus | 92.8 | 0.0052 | 0.001 |
| 12867 | 543173 | Firmicutes | Lactobacillus | 93.0 | 0.0013 | 0.001 |
| 13521 | 686900 | Firmicutes | Oribacterium | 98.8 | 0.0037 | 0.001 |
| 28350 | 770430 | Firmicutes | Other Bacillaceae | 93.0 | 0.0031 | 0.001 |
| 49543 | 624099 | Firmicutes | Other Bacillaceae | 92.3 | 0.0025 | 0.001 |
| 27889 | 45031 | Firmicutes | Other Clostridiaceae | 96.0 | 0.0023 | 0.001 |
| 19421 | 162800 | Firmicutes | Other Clostridiales | 98.0 | 0.0021 | 0.001 |
| 4517 | 4413165 | Firmicutes | Other Lachnospiraceae | 97.5 | 0.0029 | 0.001 |
| 29009 | 180707 | Firmicutes | Other Lachnospiraceae | 98.8 | 0.0022 | 0.001 |
| 19779 | 92161 | Firmicutes | Other Veillonellaceae | 91.1 | 0.0018 | 0.001 |
| 6918 | 4431592 | Firmicutes | Paenibacillus | 93.0 | 0.0007 | 0.001 |
| 16949 | 4381875 | Firmicutes | Planococcus | 93.0 | 0.0011 | 0.001 |
| 48740 | 254476 | Firmicutes | Schwartzia | 92.3 | 0.0014 | 0.001 |
| 12280 | 851782 | Firmicutes | Shuttleworthia | 97.8 | 0.0017 | 0.001 |
| 8570 | 4315958 | Firmicutes | Streptococcus | 92.3 | 0.0083 | 0.001 |
| 44302 | 897007 | Firmicutes | Streptococcus | 92.3 | 0.0008 | 0.001 |
| 5729 | 1107359 | Firmicutes | Tepidimicrobium | 98.8 | 0.0008 | 0.001 |
| 3722 | 636042 | Firmicutes | Geobacillus | 92.8 | 0.0005 | 0.002 |
| 8938 | 202367 | Firmicutes | Other Erysipelotrichaceae | 89.9 | 0.0005 | 0.002 |
| 30580 | 671376 | Firmicutes | Ureibacillus | 93.0 | 0.0209 | 0.002 |
| 22533 | 369013 | Firmicutes | Carnobacterium | 92.9 | 0.0005 | 0.003 |
| 9341 | 3851582 | Firmicutes | Lactobacillus | 92.7 | 0.0008 | 0.003 |
| 44538 | 152111 | Firmicutes | Other Bacillaceae | 92.7 | 0.0046 | 0.003 |
| 9473 | 786831 | Firmicutes | Other Bacillaceae | 91.1 | 0.0009 | 0.003 |
| 18286 | 4448492 | Firmicutes | Other Lachnospiraceae | 98.8 | 0.0007 | 0.003 |
| 10995 | 514095 | Firmicutes | Other Veillonellaceae | 90.9 | 0.0006 | 0.003 |
| 23036 | 540856 | Firmicutes | Clostridium | 97.5 | 0.0017 | 0.006 |
| 21476 | 4064550 | Firmicutes | Erysipelothrix | 89.4 | 0.0007 | 0.006 |
| 10133 | 16164 | Firmicutes | Other Clostridiaceae | 93.5 | 0.0007 | 0.006 |
| 14762 | 147321 | Firmicutes | Other Eubacteriaceae | 97.8 | 0.0005 | 0.006 |
| 15829 | 416994 | Firmicutes | Other Staphylococcaceae | 91.6 | 0.0004 | 0.006 |
| 42024 | 4415616 | Firmicutes | Bacillus | 92.8 | 0.0006 | 0.011 |
| 19373 | 1016369 | Firmicutes | Brochothrix | 76.0 | 0.0004 | 0.011 |
| 39020 | 768514 | Firmicutes | Gallicola | 98.0 | 0.0084 | 0.011 |
| 21586 | 1132622 | Firmicutes | Megasphaera | 92.5 | 0.0004 | 0.011 |
| 3170 | 219154 | Firmicutes | Other Bacillaceae | 92.8 | 0.0004 | 0.011 |
| 37362 | 16108 | Firmicutes | Caloramator | 98.3 | 0.0135 | 0.014 |
| 40108 | 14157 | Firmicutes | Eubacterium | 97.5 | 0.0010 | 0.020 |
| 21128 | 2867944 | Firmicutes | Other Peptococcaceae | 93.6 | 0.0003 | 0.020 |
| 13242 | 521996 | Firmicutes | Streptococcus | 91.8 | 0.0005 | 0.020 |
| 4058 | 173986 | Firmicutes | Faecalibacterium | 98.8 | 0.0078 | 0.023 |
| 40565 | 830659 | Firmicutes | Lactococcus | 92.7 | 0.0041 | 0.023 |
| 11407 | 548055 | Firmicutes | Bacillus | 93.0 | 0.0014 | 0.038 |
| 21865 | 534498 | Firmicutes | Bacillus | 92.8 | 0.0003 | 0.038 |
| 39230 | 84709 | Firmicutes | Lactobacillus | 92.5 | 0.0003 | 0.038 |
| 39583 | 22697 | Firmicutes | Other Veillonellaceae | 86.9 | 0.0005 | 0.038 |
| 20827 | 569215 | Firmicutes | Tepidanaerobacter | 98.0 | 0.0033 | 0.044 |
| 665 | 2234838 | Firmicutes | Clostridium | 98.3 | 0.0020 | 0.045 |
|  |  |  |  |  |  |  |
| 1056 | 65700 | OD1 | Other OD1_genera_incertae_sedis | 90.2 | 0.0018 | 0.001 |
| 37657 | 65700 | OD1 | Other OD1_genera_incertae_sedis | 90.0 | 0.0023 | 0.001 |
| 29226 | 106562 | OD1 | Other OD1_genera_incertae_sedis | 94.1 | 0.0044 | 0.001 |
| 167 | 277716 | OD1 | Other OD1_genera_incertae_sedis | 87.1 | 0.0009 | 0.001 |
| 29530 | 277716 | OD1 | Other OD1_genera_incertae_sedis | 87.7 | 0.0009 | 0.001 |
| 39942 | 277716 | OD1 | Other OD1_genera_incertae_sedis | 88.0 | 0.0014 | 0.001 |
| 24971 | 678059 | OD1 | Other OD1_genera_incertae_sedis | 83.0 | 0.0016 | 0.001 |
| 45030 | 1114884 | OD1 | Other OD1_genera_incertae_sedis | 91.6 | 0.0008 | 0.001 |
| 22766 | 2881191 | OD1 | Other OD1_genera_incertae_sedis | 90.1 | 0.0012 | 0.001 |
| 34191 | 4322208 | OD1 | Other OD1_genera_incertae_sedis | 86.0 | 0.0029 | 0.001 |
| 39661 | 4352346 | OD1 | Other OD1_genera_incertae_sedis | 87.7 | 0.0021 | 0.001 |
| 49657 | 4405880 | OD1 | Other OD1_genera_incertae_sedis | 85.1 | 0.0025 | 0.001 |
| 49722 | 4467411 | OD1 | Other OD1_genera_incertae_sedis | 88.9 | 0.0007 | 0.001 |
| 49918 | 4322208 | OD1 | Other OD1_genera_incertae_sedis | 87.9 | 0.0008 | 0.006 |
| 12559 | 4443105 | OD1 | Other OD1_genera_incertae_sedis | 93.1 | 0.0006 | 0.006 |
| 38228 | 210146 | OD1 | Other OD1_genera_incertae_sedis | 89.6 | 0.0012 | 0.011 |
| 1643 | 531358 | OD1 | Other OD1_genera_incertae_sedis | 92.1 | 0.0003 | 0.020 |
| 6148 | 531358 | OD1 | Other OD1_genera_incertae_sedis | 89.4 | 0.0005 | 0.038 |
| 12740 | 531358 | OD1 | Other OD1_genera_incertae_sedis | 92.9 | 0.0009 | 0.038 |
| 28407 | 821942 | OD1 | Other OD1_genera_incertae_sedis | 94.3 | 0.0005 | 0.038 |
| 23590 | 4310797 | OD1 | Other OD1_genera_incertae_sedis | 87.9 | 0.0006 | 0.038 |
| 48632 | 4443105 | OD1 | Other OD1_genera_incertae_sedis | 84.3 | 0.0006 | 0.038 |
| 26421 | 4304962 | OD1 | Other OD1_genera_incertae_sedis | 93.6 | 0.0035 | 0.047 |
|  |  |  |  |  |  |  |
| 19057 | 57713 | OP10 | Other OP10_genera_incertae_sedis | 94.8 | 0.0008 | 0.001 |
|  |  |  |  |  |  |  |
| 39166 | 279203 | Planctomycetes | Gemmata | 94.2 | 0.0007 | 0.001 |
| 16998 | 4365794 | Planctomycetes | Other Planctomycetaceae | 90.6 | 0.0012 | 0.001 |
| 46546 | 801268 | Planctomycetes | Singulisphaera | 93.0 | 0.0059 | 0.001 |
|  |  |  |  |  |  |  |
| 26113 | 938794 | Proteobacteria | Acinetobacter | 92.5 | 0.0049 | 0.001 |
| 4998 | 167511 | Proteobacteria | Bdellovibrio | 93.1 | 0.0021 | 0.001 |
| 41569 | 456084 | Proteobacteria | Bdellovibrio | 90.9 | 0.0013 | 0.001 |
| 16630 | 268353 | Proteobacteria | Brevundimonas | 98.3 | 0.0025 | 0.001 |
| 46787 | 114621 | Proteobacteria | Campylobacter | 98.3 | 0.0044 | 0.001 |
| 26245 | 151835 | Proteobacteria | Collimonas | 82.9 | 0.0049 | 0.001 |
| 7220 | 3768650 | Proteobacteria | Colwellia | 92.7 | 0.0014 | 0.001 |
| 3706 | 763702 | Proteobacteria | Hyphomicrobium | 97.3 | 0.0045 | 0.001 |
| 18579 | 610433 | Proteobacteria | Legionella | 92.3 | 0.0014 | 0.001 |
| 8006 | 304543 | Proteobacteria | Methylophilus | 92.7 | 0.0014 | 0.001 |
| 39975 | 4413869 | Proteobacteria | Methylophilus | 93.0 | 0.0014 | 0.001 |
| 19851 | 4364412 | Proteobacteria | Other Alphaproteobacteria | 97.5 | 0.0038 | 0.001 |
| 8070 | 203879 | Proteobacteria | Other Betaproteobacteria | 92.7 | 0.0022 | 0.001 |
| 39444 | 647595 | Proteobacteria | Other Betaproteobacteria | 93.0 | 0.0019 | 0.001 |
| 18120 | 4315533 | Proteobacteria | Other Betaproteobacteria | 89.7 | 0.0032 | 0.001 |
| 14728 | 325947 | Proteobacteria | Other Burkholderiales | 85.2 | 0.0018 | 0.001 |
| 7260 | 284413 | Proteobacteria | Other Gammaproteobacteria | 92.7 | 0.0009 | 0.001 |
| 10009 | 4042102 | Proteobacteria | Other Gammaproteobacteria | 88.3 | 0.0037 | 0.001 |
| 16014 | 1064003 | Proteobacteria | Other Gammaproteobacteria | 90.2 | 0.0031 | 0.001 |
| 23570 | 690461 | Proteobacteria | Other Myxococcales | 92.8 | 0.0032 | 0.001 |
| 27546 | 30991 | Proteobacteria | Other Myxococcales | 93.8 | 0.0012 | 0.001 |
| 6583 | 992439 | Proteobacteria | Other Proteobacteria | 92.8 | 0.0011 | 0.001 |
| 12296 | 871009 | Proteobacteria | Other Proteobacteria | 85.9 | 0.0009 | 0.001 |
| 37355 | 311522 | Proteobacteria | Other Pseudomonadales | 89.9 | 0.0021 | 0.001 |
| 22586 | 4306218 | Proteobacteria | Other Rhodospirillales | 94.8 | 0.0022 | 0.001 |
| 34135 | 156452 | Proteobacteria | Other Sphingomonadaceae | 96.0 | 0.0030 | 0.001 |
| 51236 | 3189294 | Proteobacteria | Other Sphingomonadaceae | 98.5 | 0.0035 | 0.001 |
| 28043 | 752563 | Proteobacteria | Other Sphingomonadales | 98.8 | 0.0016 | 0.001 |
| 14982 | 554390 | Proteobacteria | Peredibacter | 90.9 | 0.0024 | 0.001 |
| 2808 | 4385479 | Proteobacteria | Proteus | 92.0 | 0.0025 | 0.001 |
| 17527 | 253214 | Proteobacteria | Pseudochrobactrum | 98.8 | 0.0040 | 0.001 |
| 8026 | 4426432 | Proteobacteria | Pseudomonas | 92.3 | 0.0061 | 0.001 |
| 51234 | 813216 | Proteobacteria | Pseudomonas | 91.3 | 0.0008 | 0.001 |
| 11585 | 4343884 | Proteobacteria | Roseomonas | 98.8 | 0.0119 | 0.001 |
| 19177 | 4336437 | Proteobacteria | Thauera | 92.5 | 0.0029 | 0.001 |
| 47209 | 4393358 | Proteobacteria | Vibrio | 93.0 | 0.0040 | 0.001 |
| 29797 | 4315253 | Proteobacteria | Bacteriovorax | 91.8 | 0.0007 | 0.002 |
| 39509 | 1133769 | Proteobacteria | Moritella | 94.2 | 0.0016 | 0.002 |
| 47808 | 830893 | Proteobacteria | Other Rhizobiales | 97.5 | 0.0009 | 0.002 |
| 11109 | 284672 | Proteobacteria | Yersinia | 92.7 | 0.0005 | 0.002 |
| 86 | 818927 | Proteobacteria | Colwellia | 93.0 | 0.0009 | 0.003 |
| 37059 | 4323718 | Proteobacteria | Comamonas | 90.9 | 0.0009 | 0.003 |
| 36646 | 813110 | Proteobacteria | Other Alphaproteobacteria | 93.5 | 0.0005 | 0.003 |
| 23207 | 1117661 | Proteobacteria | Other Deltaproteobacteria | 87.9 | 0.0015 | 0.003 |
| 45296 | 559204 | Proteobacteria | Other Enterobacteriaceae | 91.8 | 0.0005 | 0.003 |
| 24847 | 4386920 | Proteobacteria | Pseudomonas | 92.5 | 0.0005 | 0.003 |
| 41831 | 3974550 | Proteobacteria | Shewanella | 90.4 | 0.0005 | 0.003 |
| 12128 | 948499 | Proteobacteria | Other Gammaproteobacteria | 89.7 | 0.0007 | 0.006 |
| 46710 | 3974550 | Proteobacteria | Other Gammaproteobacteria | 89.5 | 0.0004 | 0.006 |
| 16723 | 228868 | Proteobacteria | Other Proteobacteria | 92.6 | 0.0006 | 0.006 |
| 46574 | 279948 | Proteobacteria | Pseudomonas | 92.5 | 0.0017 | 0.006 |
| 5653 | 246864 | Proteobacteria | Psychrobacter | 90.2 | 0.0004 | 0.006 |
| 15231 | 143326 | Proteobacteria | Rheinheimera | 92.5 | 0.0007 | 0.006 |
| 33200 | 811982 | Proteobacteria | Oceanisphera | 92.7 | 0.0004 | 0.011 |
| 4207 | 280459 | Proteobacteria | Pseudomonas | 80.1 | 0.0008 | 0.011 |
| 23331 | 4386920 | Proteobacteria | Pseudomonas | 91.8 | 0.0004 | 0.011 |
| 1005 | 110450 | Proteobacteria | Psychrobacter | 81.9 | 0.0004 | 0.011 |
| 15824 | 68622 | Proteobacteria | Achromobacter | 95.4 | 0.0005 | 0.020 |
| 46457 | 1142199 | Proteobacteria | Ignatzschineria | 92.1 | 0.0005 | 0.020 |
| 43896 | 533038 | Proteobacteria | Methylophilus | 91.1 | 0.0003 | 0.020 |
| 17724 | 284090 | Proteobacteria | Pseudomonas | 92.5 | 0.0004 | 0.020 |
| 30283 | 4327501 | Proteobacteria | Pseudomonas | 93.0 | 0.0004 | 0.020 |
| 48862 | 3974550 | Proteobacteria | Shewanella | 90.6 | 0.0003 | 0.020 |
| 33812 | 4386920 | Proteobacteria | Pseudomonas | 91.1 | 0.0003 | 0.038 |
| 32748 | 51114 | Proteobacteria | Psychrobacter | 95.5 | 0.0003 | 0.038 |
| 20403 | 3974550 | Proteobacteria | Shewanella | 90.9 | 0.0003 | 0.038 |
| 43966 | 3974550 | Proteobacteria | Shewanella | 89.8 | 0.0003 | 0.038 |
| 1058 | 546186 | Proteobacteria | Arcobacter | 98.8 | 0.0090 | 0.045 |
|  |  |  |  |  |  |  |
| 40920 | 717486 | Spirochaetes | Treponema | 91.3 | 0.0023 | 0.001 |
| 11834 | 4017244 | Spirochaetes | Turneriella | 90.6 | 0.0012 | 0.001 |
|  |  |  |  |  |  |  |
| 4945 | 568911 | SR1 | Other SR1_genera_incertae_sedis | 94.0 | 0.0009 | 0.001 |
| 18430 | 568911 | SR1 | Other SR1_genera_incertae_sedis | 95.3 | 0.0009 | 0.003 |
|  |  |  |  |  |  |  |
| 34480 | 113976 | TM7 | Other TM7_genera_incertae_sedis | 97.0 | 0.0017 | 0.001 |
| 41257 | 165179 | TM7 | Other TM7_genera_incertae_sedis | 90.3 | 0.0026 | 0.001 |
| 18548 | 195489 | TM7 | Other TM7_genera_incertae_sedis | 95.3 | 0.0053 | 0.001 |
| 11318 | 832399 | TM7 | Other TM7_genera_incertae_sedis | 97.3 | 0.0051 | 0.001 |
| 42655 | 1887417 | TM7 | Other TM7_genera_incertae_sedis | 97.8 | 0.0029 | 0.001 |
|  |  |  |  |  |  |  |
| 47797 | 89151 | Unclassified Bacteria | Unclassified Bacteria | 94.6 | 0.0023 | 0.001 |
| 48948 | 104773 | Unclassified Bacteria | Unclassified Bacteria | 80.7 | 0.0013 | 0.001 |
| 35695 | 144545 | Unclassified Bacteria | Unclassified Bacteria | 96.0 | 0.0018 | 0.001 |
| 20461 | 205209 | Unclassified Bacteria | Unclassified Bacteria | 92.9 | 0.0023 | 0.001 |
| 32675 | 213358 | Unclassified Bacteria | Unclassified Bacteria | 84.4 | 0.0018 | 0.001 |
| 23867 | 225697 | Unclassified Bacteria | Unclassified Bacteria | 87.6 | 0.0063 | 0.001 |
| 5551 | 226009 | Unclassified Bacteria | Unclassified Bacteria | 88.1 | 0.0030 | 0.001 |
| 15650 | 278587 | Unclassified Bacteria | Unclassified Bacteria | 89.7 | 0.0017 | 0.001 |
| 51506 | 513869 | Unclassified Bacteria | Unclassified Bacteria | 87.4 | 0.0008 | 0.001 |
| 17638 | 549233 | Unclassified Bacteria | Unclassified Bacteria | 94.8 | 0.0034 | 0.001 |
| 50360 | 576700 | Unclassified Bacteria | Unclassified Bacteria | 88.8 | 0.0011 | 0.001 |
| 31244 | 589190 | Unclassified Bacteria | Unclassified Bacteria | 92.0 | 0.0018 | 0.001 |
| 50910 | 590013 | Unclassified Bacteria | Unclassified Bacteria | 97.5 | 0.0016 | 0.001 |
| 31998 | 699706 | Unclassified Bacteria | Unclassified Bacteria | 87.6 | 0.0006 | 0.001 |
| 41909 | 757820 | Unclassified Bacteria | Unclassified Bacteria | 93.8 | 0.0025 | 0.001 |
| 5295 | 1124746 | Unclassified Bacteria | Unclassified Bacteria | 92.6 | 0.0012 | 0.001 |
| 23165 | 1131894 | Unclassified Bacteria | Unclassified Bacteria | 80.1 | 0.0014 | 0.001 |
| 43812 | 2760486 | Unclassified Bacteria | Unclassified Bacteria | 94.4 | 0.0040 | 0.001 |
| 42175 | 2881190 | Unclassified Bacteria | Unclassified Bacteria | 92.6 | 0.0009 | 0.001 |
| 2253 | 3240079 | Unclassified Bacteria | Unclassified Bacteria | 92.3 | 0.0034 | 0.001 |
| 8605 | 3464844 | Unclassified Bacteria | Unclassified Bacteria | 94.6 | 0.0629 | 0.001 |
| 8377 | 3825731 | Unclassified Bacteria | Unclassified Bacteria | 87.8 | 0.0009 | 0.001 |
| 39445 | 4304962 | Unclassified Bacteria | Unclassified Bacteria | 85.3 | 0.0016 | 0.001 |
| 40652 | 4314310 | Unclassified Bacteria | Unclassified Bacteria | 82.9 | 0.0008 | 0.001 |
| 15021 | 4352346 | Unclassified Bacteria | Unclassified Bacteria | 86.7 | 0.0026 | 0.001 |
| 12949 | 4375764 | Unclassified Bacteria | Unclassified Bacteria | 93.0 | 0.0011 | 0.001 |
| 1609 | 4464974 | Unclassified Bacteria | Unclassified Bacteria | 92.6 | 0.0009 | 0.001 |
| 11258 | 4464974 | Unclassified Bacteria | Unclassified Bacteria | 90.6 | 0.0019 | 0.001 |
| 13217 | 4477135 | Unclassified Bacteria | Unclassified Bacteria | 91.3 | 0.0016 | 0.001 |
| 44130 | 827300 | Unclassified Bacteria | Unclassified Bacteria | 91.8 | 0.0007 | 0.002 |
| 30820 | 1145388 | Unclassified Bacteria | Unclassified Bacteria | 97.5 | 0.0007 | 0.002 |
| 7097 | 2927689 | Unclassified Bacteria | Unclassified Bacteria | 94.3 | 0.0011 | 0.002 |
| 32271 | 3407358 | Unclassified Bacteria | Unclassified Bacteria | 84.4 | 0.0010 | 0.002 |
| 9983 | 3746871 | Unclassified Bacteria | Unclassified Bacteria | 94.3 | 0.0010 | 0.002 |
| 37769 | 4464974 | Unclassified Bacteria | Unclassified Bacteria | 91.6 | 0.0009 | 0.002 |
| 30631 | 175067 | Unclassified Bacteria | Unclassified Bacteria | 89.7 | 0.0009 | 0.003 |
| 1017 | 829415 | Unclassified Bacteria | Unclassified Bacteria | 97.8 | 0.0009 | 0.003 |
| 11513 | 1793401 | Unclassified Bacteria | Unclassified Bacteria | 89.1 | 0.0005 | 0.003 |
| 47740 | 2927689 | Unclassified Bacteria | Unclassified Bacteria | 95.5 | 0.0008 | 0.003 |
| 19715 | 3390949 | Unclassified Bacteria | Unclassified Bacteria | 82.3 | 0.0005 | 0.003 |
| 31193 | 4415437 | Unclassified Bacteria | Unclassified Bacteria | 92.3 | 0.0008 | 0.003 |
| 46717 | 3225199 | Unclassified Bacteria | Unclassified Bacteria | 93.8 | 0.0015 | 0.005 |
| 18451 | 200762 | Unclassified Bacteria | Unclassified Bacteria | 83.3 | 0.0004 | 0.006 |
| 12285 | 3746871 | Unclassified Bacteria | Unclassified Bacteria | 95.5 | 0.0009 | 0.006 |
| 34394 | 508877 | Unclassified Bacteria | Unclassified Bacteria | 89.3 | 0.0004 | 0.011 |
| 24081 | 509699 | Unclassified Bacteria | Unclassified Bacteria | 93.5 | 0.0006 | 0.011 |
| 2746 | 3225199 | Unclassified Bacteria | Unclassified Bacteria | 95.3 | 0.0007 | 0.011 |
| 3141 | 3225199 | Unclassified Bacteria | Unclassified Bacteria | 94.6 | 0.0008 | 0.011 |
| 38469 | 3225199 | Unclassified Bacteria | Unclassified Bacteria | 94.0 | 0.0007 | 0.011 |
| 45667 | 3225199 | Unclassified Bacteria | Unclassified Bacteria | 95.0 | 0.0007 | 0.011 |
| 40436 | 4342702 | Unclassified Bacteria | Unclassified Bacteria | 88.6 | 0.0004 | 0.011 |
| 4974 | 4344792 | Unclassified Bacteria | Unclassified Bacteria | 83.4 | 0.0005 | 0.011 |
| 44204 | 4352346 | Unclassified Bacteria | Unclassified Bacteria | 84.4 | 0.0006 | 0.011 |
| 31658 | 807173 | Unclassified Bacteria | Unclassified Bacteria | 98.0 | 0.0107 | 0.013 |
| 6017 | 3225199 | Unclassified Bacteria | Unclassified Bacteria | 88.0 | 0.0195 | 0.018 |
| 50715 | 179710 | Unclassified Bacteria | Unclassified Bacteria | 82.5 | 0.0007 | 0.020 |
| 51210 | 339137 | Unclassified Bacteria | Unclassified Bacteria | 90.0 | 0.0010 | 0.020 |
| 25765 | 509699 | Unclassified Bacteria | Unclassified Bacteria | 91.8 | 0.0013 | 0.020 |
| 36169 | 2850541 | Unclassified Bacteria | Unclassified Bacteria | 95.5 | 0.0004 | 0.020 |
| 21609 | 3225199 | Unclassified Bacteria | Unclassified Bacteria | 95.3 | 0.0009 | 0.020 |
| 37426 | 3225199 | Unclassified Bacteria | Unclassified Bacteria | 94.8 | 0.0006 | 0.020 |
| 28934 | 3746871 | Unclassified Bacteria | Unclassified Bacteria | 95.0 | 0.0009 | 0.020 |
| 34003 | 4386317 | Unclassified Bacteria | Unclassified Bacteria | 90.3 | 0.0004 | 0.020 |
| 42177 | 4464974 | Unclassified Bacteria | Unclassified Bacteria | 90.4 | 0.0007 | 0.020 |
| 4803 | 521023 | Unclassified Bacteria | Unclassified Bacteria | 83.0 | 0.0004 | 0.038 |
| 6567 | 2927689 | Unclassified Bacteria | Unclassified Bacteria | 94.1 | 0.0005 | 0.038 |
| 42079 | 3225199 | Unclassified Bacteria | Unclassified Bacteria | 95.5 | 0.0007 | 0.038 |
| 32323 | 4328888 | Unclassified Bacteria | Unclassified Bacteria | 90.0 | 0.0011 | 0.038 |
|  |  |  |  |  |  |  |
| 11695 | na | Unclassified Bacteria | Unclassified Bacteria | na | 0.0013 | 0.001 |
| 13793 | na | Unclassified Bacteria | Unclassified Bacteria | na | 0.0030 | 0.001 |
| 17326 | na | Unclassified Bacteria | Unclassified Bacteria | na | 0.0034 | 0.001 |
| 3238 | na | Unclassified Bacteria | Unclassified Bacteria | na | 0.0007 | 0.002 |
| 8249 | na | Unclassified Bacteria | Unclassified Bacteria | na | 0.0007 | 0.002 |
| 9328 | na | Unclassified Bacteria | Unclassified Bacteria | na | 0.0009 | 0.003 |
| 14717 | na | Unclassified Bacteria | Unclassified Bacteria | na | 0.0007 | 0.003 |
| 33760 | na | Unclassified Bacteria | Unclassified Bacteria | na | 0.0007 | 0.003 |
| 862 | na | Unclassified Bacteria | Unclassified Bacteria | na | 0.0004 | 0.006 |
| 1133 | na | Unclassified Bacteria | Unclassified Bacteria | na | 0.0006 | 0.006 |
| 42239 | na | Unclassified Bacteria | Unclassified Bacteria | na | 0.0005 | 0.006 |
| 51455 | na | Unclassified Bacteria | Unclassified Bacteria | na | 0.0017 | 0.006 |
| 48898 | na | Unclassified Bacteria | Unclassified Bacteria | na | 0.0014 | 0.009 |
| 20136 | na | Unclassified Bacteria | Unclassified Bacteria | na | 0.0005 | 0.011 |
| 22787 | na | Unclassified Bacteria | Unclassified Bacteria | na | 0.0005 | 0.011 |
| 27629 | na | Unclassified Bacteria | Unclassified Bacteria | na | 0.0007 | 0.011 |
| 44404 | na | Unclassified Bacteria | Unclassified Bacteria | na | 0.0006 | 0.011 |
| 47879 | na | Unclassified Bacteria | Unclassified Bacteria | na | 0.0005 | 0.011 |
| 18002 | na | Unclassified Bacteria | Unclassified Bacteria | na | 0.0005 | 0.020 |
| 40538 | na | Unclassified Bacteria | Unclassified Bacteria | na | 0.0013 | 0.020 |
| 1638 | na | Unclassified Bacteria | Unclassified Bacteria | na | 0.0011 | 0.038 |
| 13348 | na | Unclassified Bacteria | Unclassified Bacteria | na | 0.0006 | 0.038 |
| 16683 | na | Unclassified Bacteria | Unclassified Bacteria | na | 0.0004 | 0.038 |
| 21537 | na | Unclassified Bacteria | Unclassified Bacteria | na | 0.0006 | 0.038 |
| 27710 | na | Unclassified Bacteria | Unclassified Bacteria | na | 0.0005 | 0.038 |
| 29429 | na | Unclassified Bacteria | Unclassified Bacteria | na | 0.0004 | 0.038 |
| 32144 | na | Unclassified Bacteria | Unclassified Bacteria | na | 0.0006 | 0.038 |
| 41805 | na | Unclassified Bacteria | Unclassified Bacteria | na | 0.0005 | 0.038 |
| 48123 | na | Unclassified Bacteria | Unclassified Bacteria | na | 0.0004 | 0.038 |
| 50026 | na | Unclassified Bacteria | Unclassified Bacteria | na | 0.0005 | 0.038 |
| 42139 | na | Unclassified Bacteria | Unclassified Bacteria | na | 0.0024 | 0.047 |
|  |  |  |  |  |  |  |
| 5317 | na | Unclassified Sequence | Unclassified Sequence | na | 0.0012 | 0.003 |
| 35657 | na | Unclassified Sequence | Unclassified Sequence | na | 0.0009 | 0.003 |
| 39671 | na | Unclassified Sequence | Unclassified Sequence | na | 0.0009 | 0.003 |
| 49002 | na | Unclassified Sequence | Unclassified Sequence | na | 0.0007 | 0.006 |
| 9736 | na | Unclassified Sequence | Unclassified Sequence | na | 0.0014 | 0.011 |
| 19321 | na | Unclassified Sequence | Unclassified Sequence | na | 0.0005 | 0.020 |

^1^ unique number assigned to sequences with less than 97% similarity to other sequences

^2^ number that is associated with the sequence in the aligned Greengenes database that fits the query sequence

^3^ percent identity of the query sequence to the assigned Greengenes reference sequence

**Supplementary table 3: Full list of Operational Taxonomic Units (OTUs) found at significantly different abundances in the distal intestine of Atlantic salmon (*Salmo salar*) parr kept in a recirculating aquarium facility and the distal intestine of Atlantic salmon (*Salmo salar*) parr kept in an open loch system, as identified by Metastats analysis.**

| **denovo^1^** | **Greengenes ID^2^** | **Phylum** | **Genus** | **BLAST % identity ^3^** | **mean abundance aqua (%)** | **mean abundance loch (%)** | **pvalue** |
| --- | --- | --- | --- | --- | --- | --- | --- |
| 38715 | 951711 | Actinobacteria | Actinomyces | 93.8 | 0.00268 | 0.02349 | 0.040 |
|  |  |  |  |  |  |  |  |
| 12987 | 4154872 | Bacteroidetes | Cloacibacterium | 93.8 | 0.02542 | 0.00194 | 0.012 |
| 5990 | 807860 | Bacteroidetes | Chryseobacterium | 92.2 | 0.00010 | 0.02127 | 0.024 |
| 41956 | 568419 | Bacteroidetes | Other Flavobacteriaceae | 93.6 | 0.00340 | 0.39807 | 0.041 |
|  |  |  |  |  |  |  |  |
| 18978 | 572843 | Firmicutes | Granulicatella | 93.0 | 0.00088 | 0.05300 | 0.001 |
| 11557 | 132829 | Firmicutes | Lactobacillus | 92.5 | 0.03479 | 1.01249 | 0.001 |
| 44038 | 707087 | Firmicutes | Clostridium | 98.8 | 0.00005 | 0.03993 | 0.002 |
| 15815 | 342014 | Firmicutes | Lactobacillus | 92.7 | 0.00740 | 0.12106 | 0.008 |
| 44087 | 64384 | Firmicutes | Weissella | 92.7 | 0.00705 | 0.04146 | 0.008 |
| 9412 | 921110 | Firmicutes | Vagococcus | 91.5 | 0.00005 | 0.00315 | 0.009 |
| 8290 | 518033 | Firmicutes | Lactobacillus | 93.0 | 0.00010 | 0.01992 | 0.015 |
| 34912 | 52399 | Firmicutes | Atopstipes | 92.7 | 0.00408 | 0.00004 | 0.018 |
| 8718 | 694337 | Firmicutes | Other Ruminococcaceae | 88.3 | 0.00110 | 0.00014 | 0.018 |
| 49600 | 4469032 | Firmicutes | Lactobacillus | 93.0 | 0.01466 | 0.04319 | 0.042 |
| 26115 | 3804335 | Firmicutes | Peptostreptococcus | 98.8 | 0.00346 | 0.02219 | 0.044 |
|  |  |  |  |  |  |  |  |
| 8325 | 726699 | Fusobacteria | Psychrilyobacter | 97.0 | 0.03149 | 0.09922 | 0.030 |
|  |  |  |  |  |  |  |  |
| 32094 | 346434 | OD1 | Other OD1_genera_incertae_sedis | 94.3 | 0.00005 | 0.00086 | 0.016 |
|  |  |  |  |  |  |  |  |
| 26690 | 209511 | Proteobacteria | Acinetobacter | 91.8 | 0.00826 | 0.21575 | 0.001 |
| 34063 | 3799784 | Proteobacteria | Escherichia/Shigella | 92.7 | 4.61424 | 0.18189 | 0.001 |
| 9191 | 608710 | Proteobacteria | Psychrobacter | 92.5 | 0.00227 | 0.47395 | 0.002 |
| 10383 | 1127531 | Proteobacteria | Aeromonas | 92.5 | 0.00469 | 0.14556 | 0.003 |
| 12015 | 4452489 | Proteobacteria | Photobacterium | 93.0 | 0.13725 | 0.60965 | 0.008 |
| 4538 | 324143 | Proteobacteria | Psychrobacter | 92.3 | 0.00005 | 0.00084 | 0.016 |
| 2999 | 1941457 | Proteobacteria | Photobacterium | 92.7 | 0.00241 | 0.01532 | 0.020 |
| 28037 | 106476 | Proteobacteria | Aliivibrio | 93.0 | 0.13670 | 1.62928 | 0.020 |
| 6392 | 106316 | Proteobacteria | Photobacterium | 92.7 | 0.00166 | 0.01087 | 0.023 |
| 32744 | 4352233 | Proteobacteria | Pseudomonas | 92.3 | 0.00115 | 0.03316 | 0.034 |
| 49596 | 112983 | Proteobacteria | Moritella | 92.5 | 0.31460 | 2.90082 | 0.038 |
| 7872 | 79299 | Proteobacteria | Psychrobacter | 92.3 | 0.00283 | 0.02574 | 0.040 |
|  |  |  |  |  |  |  |  |
| 47445 | 147311 | Tenericutes | Other Mycoplasmataceae | 89.7 | 14.01957 | 2.17272 | 0.002 |
|  |  |  |  |  |  |  |  |
| 14992 | 3225199 | Unclassified Bacteria | Unclassified Bacteria | 95.3 | 0.00017 | 0.00324 | 0.001 |
| 37904 | 3225199 | Unclassified Bacteria | Unclassified Bacteria | 96.0 | 0.00068 | 0.01703 | 0.001 |
| 38366 | 3225199 | Unclassified Bacteria | Unclassified Bacteria | 95.8 | 0.00064 | 0.01377 | 0.002 |
| 4291 | 3225199 | Unclassified Bacteria | Unclassified Bacteria | 94.6 | 0.00022 | 0.00403 | 0.003 |
| 34404 | 3746871 | Unclassified Bacteria | Unclassified Bacteria | 92.5 | 0.01866 | 0.00054 | 0.003 |
| 25245 | 3225199 | Unclassified Bacteria | Unclassified Bacteria | 94.8 | 0.00032 | 0.00480 | 0.004 |
| 47383 | 3225199 | Unclassified Bacteria | Unclassified Bacteria | 95.5 | 0.00016 | 0.00194 | 0.004 |
| 14388 | 3225199 | Unclassified Bacteria | Unclassified Bacteria | 94.8 | 0.00044 | 0.00265 | 0.005 |
| 42761 | 3225199 | Unclassified Bacteria | Unclassified Bacteria | 95.8 | 0.00072 | 0.01061 | 0.005 |
| 3776 | 3225199 | Unclassified Bacteria | Unclassified Bacteria | 96.3 | 0.00164 | 0.02238 | 0.006 |
| 4900 | 3225199 | Unclassified Bacteria | Unclassified Bacteria | 95.5 | 0.00035 | 0.00450 | 0.006 |
| 5517 | 3225199 | Unclassified Bacteria | Unclassified Bacteria | 94.3 | 0.00031 | 0.00393 | 0.007 |
| 12112 | 3225199 | Unclassified Bacteria | Unclassified Bacteria | 95.3 | 0.00014 | 0.00305 | 0.007 |
| 22477 | 3225199 | Unclassified Bacteria | Unclassified Bacteria | 94.3 | 0.00012 | 0.00153 | 0.007 |
| 36723 | 3225199 | Unclassified Bacteria | Unclassified Bacteria | 95.8 | 0.00034 | 0.00414 | 0.007 |
| 36927 | 3225199 | Unclassified Bacteria | Unclassified Bacteria | 94.3 | 0.00033 | 0.01375 | 0.007 |
| 45679 | 3225199 | Unclassified Bacteria | Unclassified Bacteria | 94.8 | 0.00021 | 0.00514 | 0.008 |
| 51159 | 3225199 | Unclassified Bacteria | Unclassified Bacteria | 95.8 | 0.00085 | 0.00676 | 0.008 |
| 3772 | 3225199 | Unclassified Bacteria | Unclassified Bacteria | 95.8 | 0.00052 | 0.00393 | 0.009 |
| 31129 | 3225199 | Unclassified Bacteria | Unclassified Bacteria | 95.5 | 0.00169 | 0.02131 | 0.009 |
| 32086 | 3225199 | Unclassified Bacteria | Unclassified Bacteria | 95.5 | 0.00005 | 0.00174 | 0.009 |
| 37443 | 3225199 | Unclassified Bacteria | Unclassified Bacteria | 95.8 | 0.00094 | 0.01053 | 0.010 |
| 34013 | 3225199 | Unclassified Bacteria | Unclassified Bacteria | 95.5 | 0.00029 | 0.00260 | 0.011 |
| 17366 | 3225199 | Unclassified Bacteria | Unclassified Bacteria | 95.5 | 0.00029 | 0.00339 | 0.014 |
| 46636 | 3225199 | Unclassified Bacteria | Unclassified Bacteria | 95.8 | 0.00060 | 0.00623 | 0.015 |
| 20742 | 3225199 | Unclassified Bacteria | Unclassified Bacteria | 95.3 | 0.00008 | 0.00133 | 0.016 |
| 26850 | 3225199 | Unclassified Bacteria | Unclassified Bacteria | 94.6 | 0.00014 | 0.00188 | 0.018 |
| 38607 | 3225199 | Unclassified Bacteria | Unclassified Bacteria | 95.3 | 0.00096 | 0.01041 | 0.019 |
| 48132 | 3225199 | Unclassified Bacteria | Unclassified Bacteria | 95.5 | 0.00041 | 0.00372 | 0.020 |
| 26741 | 3746876 | Unclassified Bacteria | Unclassified Bacteria | 94.3 | 0.00050 | 0.00015 | 0.025 |
| 12806 | 3746871 | Unclassified Bacteria | Unclassified Bacteria | 90.2 | 0.01665 | 0.00168 | 0.026 |
| 46264 | 3225199 | Unclassified Bacteria | Unclassified Bacteria | 94.3 | 0.00095 | 0.00555 | 0.027 |
| 19144 | 3746871 | Unclassified Bacteria | Unclassified Bacteria | 92.9 | 0.00005 | 0.00077 | 0.029 |
| 17651 | 3225199 | Unclassified Bacteria | Unclassified Bacteria | 95.8 | 0.00008 | 0.00132 | 0.036 |
| 40626 | 3225199 | Unclassified Bacteria | Unclassified Bacteria | 94.6 | 0.00036 | 0.00426 | 0.038 |
| 22343 | 3225199 | Unclassified Bacteria | Unclassified Bacteria | 96.0 | 0.00050 | 0.00356 | 0.039 |
| 4618 | 147311 | Unclassified Bacteria | Unclassified Bacteria | 88.3 | 0.00142 | 0.00009 | 0.041 |
| 48483 | 4420570 | Unclassified Bacteria | Unclassified Bacteria | 90.8 | 0.00024 | 0.01614 | 0.041 |
| 21296 | 3746871 | Unclassified Bacteria | Unclassified Bacteria | 94.6 | 0.00211 | 0.00007 | 0.044 |
| 39118 | 3225199 | Unclassified Bacteria | Unclassified Bacteria | 95.8 | 0.00010 | 0.00117 | 0.049 |
|  |  |  |  |  |  |  |  |
| 16911 | na | Unclassified Bacteria | Unclassified Bacteria | na | 0.00007 | 0.00071 | 0.029 |

^1^ unique number assigned to sequences with less than 97% similarity to other sequences

^2^ number that is associated with the sequence in the aligned Greengenes database that fits the query sequence

^3^ percent identity of the query sequence to the assigned Greengenes reference sequence
